# Co-culture of human AT2 cells with fibroblasts reveals a MUC5B phenotype: insights from an organoid model

**DOI:** 10.1186/s10020-024-00990-w

**Published:** 2024-11-23

**Authors:** Yiwen Yao, Felix Ritzmann, Sarah Miethe, Kathrin Kattler-Lackes, Betül Colakoglu, Christian Herr, Andreas Kamyschnikow, Michelle Brand, Holger Garn, Daniela Yildiz, Frank Langer, Robert Bals, Christoph Beisswenger

**Affiliations:** 1 Department of Internal Medicine V – Pulmonology, Allergology and Critical Care Medicine, Saarland University, 66421 Homburg, Germany; 2 Department of Clinical Medicine, Shanghai Tongji Hospital, School of Medicine, Tongji University, 200065 Shanghai, China; 3 Translational Inflammation Research Division & Core Facility for Single Cell Multiomics, Medical Faculty, Member of the German Center for Lung Research (DZL) and the Universities of Giessen and Marburg Lung Center, Philipps University of Marburg, D-35043 Marburg, Germany; 4 Department of Genetics/Epigenetics, Saarland University, 66123 Saarbrücken, Germany; 5 Department of Drug Delivery (DDEL), Helmholtz-Institute for Pharmaceutical Research Saarland (HIPS), Helmholtz Centre for Infection Research (HZI), Saarbrücken, Germany; 6 Experimental and Clinical Pharmacology and Toxicology, PZMS, Saarland University, 66421 Homburg, Germany; 7 Department of Thoracic- and Cardiovascular Surgery, Saarland University Hospital, Homburg/Saar, Germany

**Keywords:** Organoid, Pneumocyte, Fibroblast, STAT3, IPF

## Abstract

Impaired interaction of fibroblasts with pneumocytes contributes to the progression of chronic lung disease such as idiopathic pulmonary fibrosis (IPF). Mucin 5B (MUC5B) is associated with IPF. Here we analyzed the interaction of primary fibroblasts and alveolar type 2 (AT2) pneumocytes in the organoid model. Single-cell analysis, histology, and qRT-PCR revealed that fibroblasts expressing high levels of fibrosis markers regulate STAT3 signaling in AT2 cells, which is accompanied by cystic organoid growth and MUC5B expression. Cystic growth and MUC5B expression were also caused by the cytokine IL-6. The PI3K-Akt signaling pathway was activated in fibroblasts. The drug dasatinib prevented the formation of MUC5B-expressing cystic organoids. MUC5B associated with AT2 cells in samples obtained from IPF patients. Our model shows that fibrotic primary fibroblasts induce impaired differentiation of AT2 cells via STAT3 signaling pathways, as observed in IPF patients. It can be used for mechanistic studies and drug development.

**Supplementary Information:**

The online version contains supplementary material available at 10.1186/s10020-024-00990-w.

## Introduction

Gas exchange in the lung takes place in millions of alveoli. The alveolar epithelium consists of cuboidal alveolar type 2 (AT2) pneumocytes, which secrete various factors such as surface tension-lowering surfactants, and flat AT1 pneumocytes, which make up most of the alveolar surface for gas exchange (Milad and Morissette 2021). The proper interaction of the pneumocytes with numerous other cell types such as alveolar fibroblasts is essential for normal lung homeostasis but also for the regeneration of the lung epithelium after, for example, infections. The stem cell properties of AT2 cells are particularly important here (Juul et al. 2020). Thus, various studies have investigated how mesenchymal cells regulate the proliferation and differentiation of lung epithelial cells (Yao et al. 2024; Kathiriya et al. 2022; Murthy et al. 2022; Alysandratos et al. 2022; Lee et al. 2017; Tsukui et al. 2024; Barkauskas et al. 2013; Zepp et al. 2017) and, conversely, how epithelial cells influence fibroblasts (Yao et al. 2024; Murthy et al. 2022; Lee et al. 2017; Ushakumary et al. 2021). For example, at least in mice, fibroblasts mediate the growth, self-renewal and differentiation of AT2 progenitor cells via the wingless-related integration site (WNT)-, fibroblast growth factors (FGF)- and IL-6/activator of transcription-3 (STAT3)-regulated ways (Yao et al. 2024; Lee et al. 2017; Zepp et al. 2017; Ushakumary et al. 2021; Riccetti et al. 2020). A central role of the Wnt and Fgf pathways has also been demonstrated in the differentiation of AT2 cells in interaction with fibroblasts in human organoids and by analysis of patient samples (Zacharias et al. 2018; Aros et al. 2021; Jacob et al. 2017; Konigshoff et al. 2009).

However, defective AT2 and mesenchymal cells are also associated with the pathogenesis of chronic lung disease such as chronic obstructive pulmonary disease (COPD) and idiopathic pulmonary fibrosis (IPF). These diseases are characterized by a massive loss of lung function and are incurable. In IPF, scarring of the lungs occurs due to fibrotic, overactivated fibroblasts and a loss of pneumocytes (Ushakumary et al. 2021; Parimon et al. 2020; Tsukui et al. 2020). COPD is characterized by chronic pulmonary inflammation and parenchymal destruction leading to emphysema (Guarnier et al. 2023). It is likely that fibroblasts contribute to the ongoing inflammation in the lungs of COPD patients by secreting inflammatory and senescence-related factors (Woldhuis et al. 2021; Ghonim et al. 2023). However, the extent to which impaired fibroblast interaction with AT2 cells leads to lung parenchymal loss remains unclear.

The occurrence of IPF is associated with a variety of risk factors, including genetic risks such as certain MUC5B promoter variants, environmental exposures (e.g. smoking, viral infections), and aging (Michalski and Schwartz 2020). Repeated microinjuries to the alveolar epithelium are thought to play a role in the development of IPF. These injuries potentially contribute to impaired communication between epithelial cells and fibroblasts, leading to activation and proliferation of misdirected fibroblasts, accumulation of large amounts of extracellular matrix (ECM), remodeling of the ECM, cellular senescence, and destruction of the lung epithelium (Martinez et al. 2017). Signaling pathways important for normal lung homeostasis, such as Wnt-signaling, are thought to play a critical role in the progression of IPF when imbalanced (Aros et al. 2021; Ye and Hu 2021).

The aim of the present study was to analyze the interaction of primary human fibroblasts and AT2 cells in an organoid model. We show that co-cultivation of AT2 organoids with fibrotic fibroblasts leads to STAT3 activation and aberrant secretory activity characterized e.g. by MUC5B. Fibroblasts express factors that activate STAT3 pathways in AT2 cells and IL-6 induces cystic growth of the organoids.

## Results

### Co-culture with fibroblasts leads to cystic growth of the organoids

**Fig. 1:**
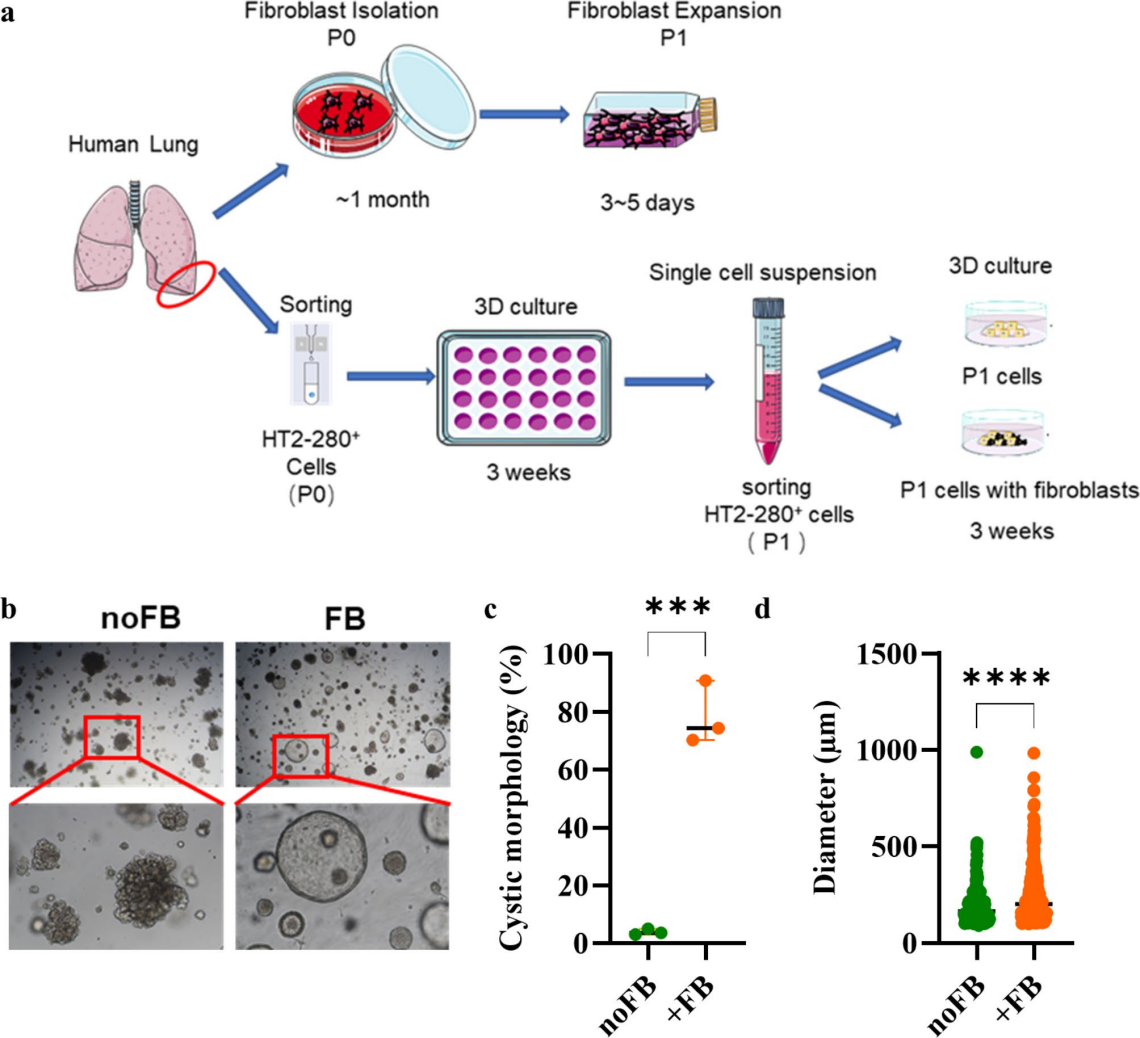
Fibroblasts induce cystic organoid growth. (**a**) Scheme of the experimental layout. (**b**) Representative phase contrast images of organoids cultured for 21 days (noFB: AT2 cells cultured without fibroblasts (FB); FB: AT2 cells cultured in the presence of fibroblasts, P1: passage 1). (**c**) Quantification of the morphology of the alveolar organoids. Data were compared by unpaired t-test (***p < 0.001). Each data point represents an independent experiment. (**d**) Quantification of the diameter of the alveolar organoids. Pooled results from 3 independent experiments. Data were compared by Mann-Whitney test (****p < 0.0001)

To investigate the impact of fibroblasts on alveolar organoid differentiation, we isolated AT2 cells and differentiated them in co-culture with primary fibroblasts for 21 days or control media (Fig. 1a). Immunostaining confirmed the AT2 identity of the cells as well as the absence of the airway epithelial markers KRT5 and CCSP (S1). Co-cultivation with fibroblasts significantly influenced the morphology of the organoids. In the absence of fibroblasts, more than 95% of the organoids showed a grape-like growth, while organoids cultured with fibroblasts had a cystic morphology (Fig. 1b and c). This was accompanied by an increased diameter of the organoids co-cultured with fibroblasts (Fig. 1d). Co-culture with the fibroblast cell line MRC5 led to larger organoids, but not to a cystic growth comparable to primary fibroblasts (S2).

### Fibroblasts induce a secretory phenotype in alveolar type-2 cells

**Fig. 2:**
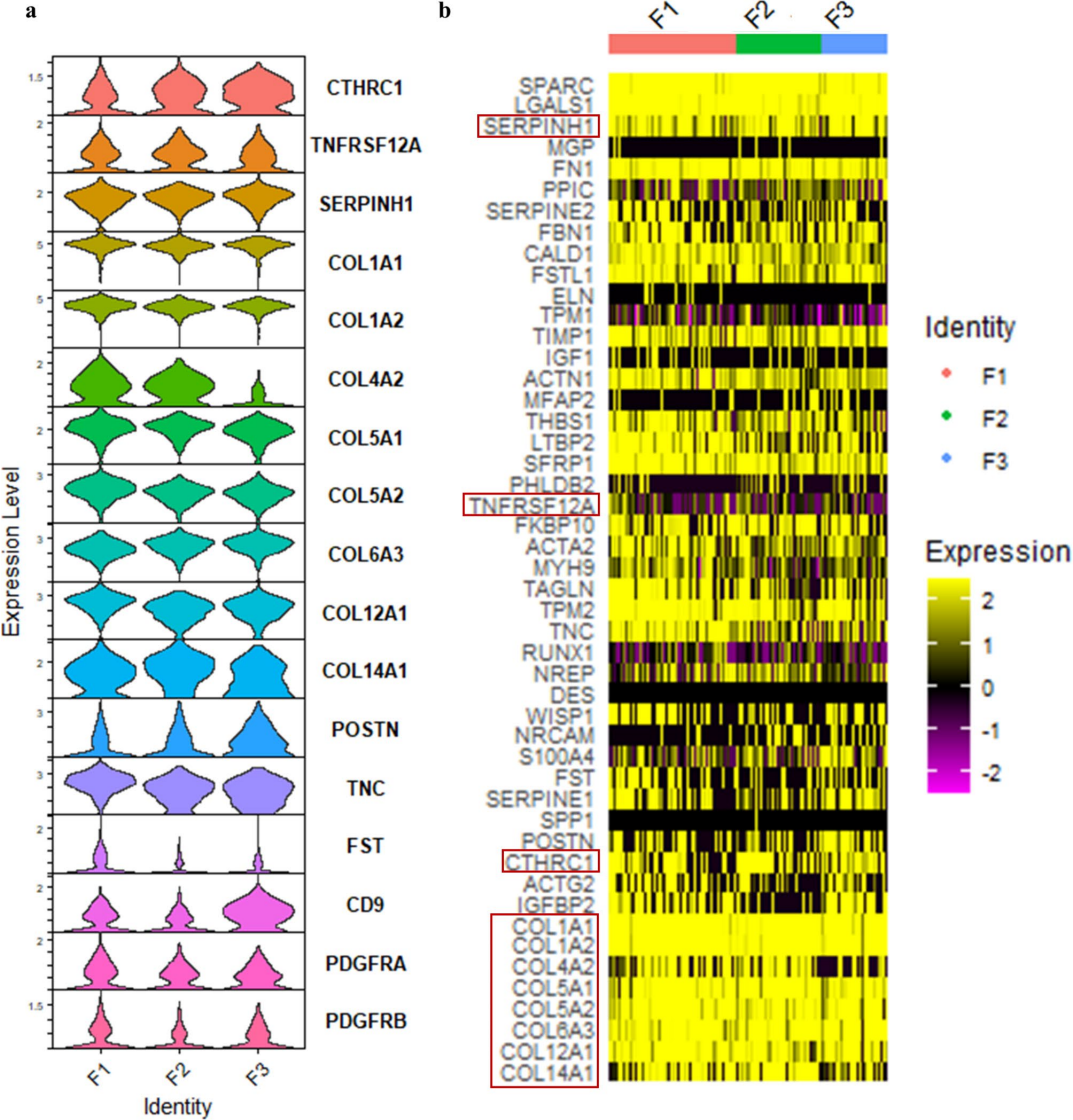
Fibroblasts show a pro-fibrotic phenotype. (**a**) Violin plots showing the expression levels of fibrosis markers for the three donors (F1-3). (**b**) Heatmap for markers of pathogenic fibroblasts. Key pro-fibrotic genes are highlighted

Next, we characterized alveolar organoids in co-culture with fibroblasts obtained from three different donors for 21 days by scRNA sequencing using BD Rhapsody™ Single-Cell Analysis System. Representative markers (Sikkema et al. 2023) showed that the cultures were composed of epithelial cells (EPCAM) and fibroblasts (COL1A1), with the fibroblasts only being found in co-cultures (S3). The fibroblasts expressed typical fibroblast markers, high levels of collagens as well as markers specifically produced by fibrotic fibroblasts such as CTHRC1, SERPINH1 and TNFRSF12A (Fig. 2) (Tsukui et al. 2024; Peyser et al. 2019; Herrera et al. 2022; Guo et al. 2024). Since the fibroblasts were isolated from disease-free regions of the lung (F1: lung cancer; F2: lung cancer with COPD; F3: pulmonary fibrosis), the phenotype of the fibroblasts is model-related and independent of the donor.

**Fig. 3:**
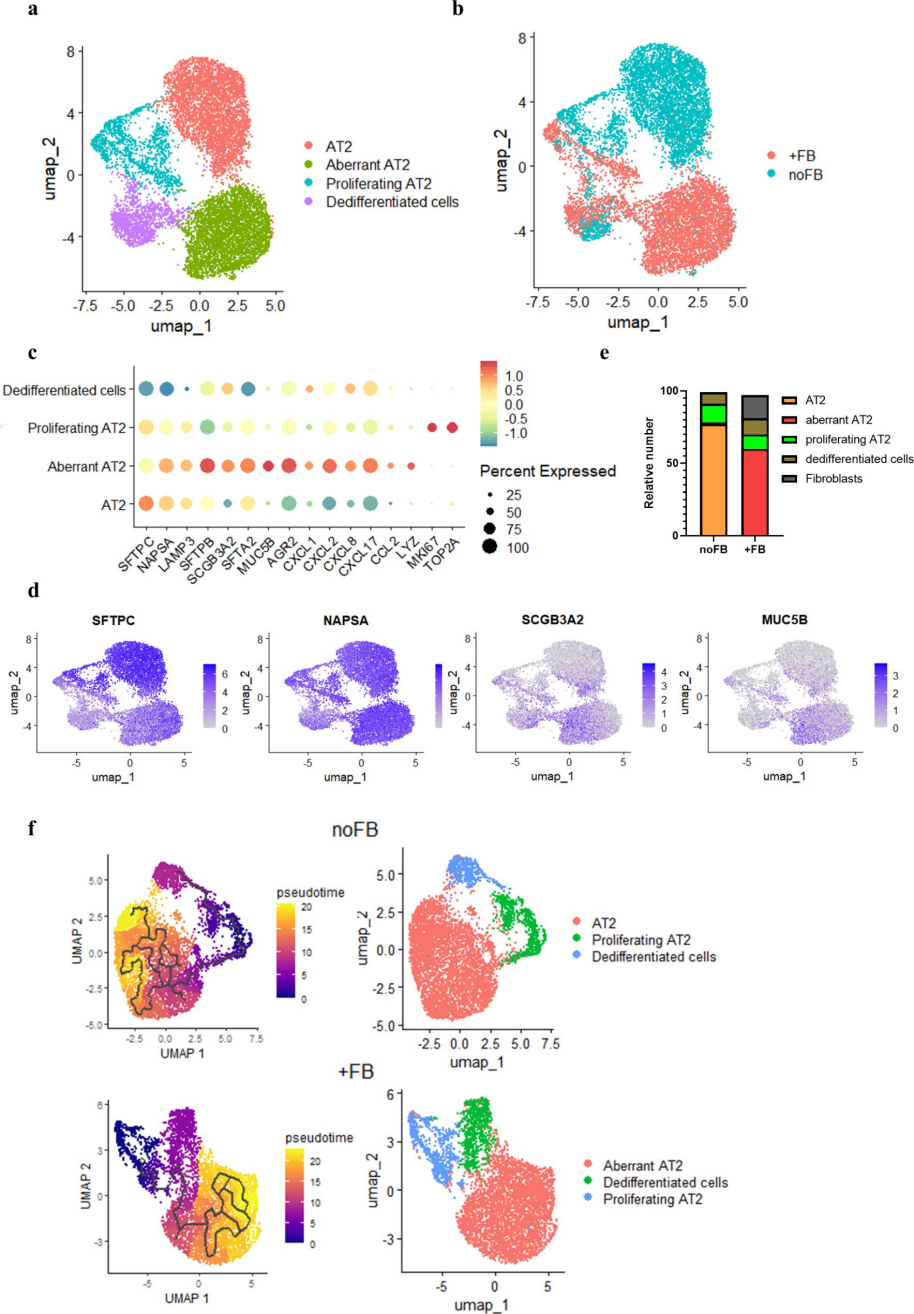
Fibroblasts drive the differentiation of AT2 cells toward a secretory phenotype. (**a**) UMAP visualization of different cell types. (**b**) UMAP visualization colored by groups (noFB: AT2 cells cultured without fibroblasts; +FB: AT2 cells cultured in the presence of fibroblasts). (**c**) Dot plot showing expression of epithelial cell type markers and secretory factors markers. (**d**) Feature plots showing the expression of representative markers for AT2 and secretory cells. (**e**) Proportion of the different cell clusters. (**f**) Pseudotime trajectory analysis by Monocle 3 of the two groups

For further analysis, we sub-clustered the epithelial cells and identified 4 clusters: AT2 cells, aberrant AT2 cells, proliferating AT2 cells, and dedifferentiated cells (Fig. 3a to c). Aberrant AT2 showed reduced expression of SFTPC, while other type 2 markers such as NAPSA and LAMP3 were increased. In addition, aberrant AT2 expressed secretory factors such as MUC5B and CXCL8. Dedifferentiated cells showed low expression for SFTPC and high expression for SFTPB and SCGB3A2 and resembled the recently discovered human terminal and respiratory bronchiole secretory cells (TRB-SCs) , which can differentiate from AT2 cells (Fig. 3c and d, S4a/b) (Murthy et al. 2022). Co-culture with fibroblasts resulted in a shift from AT2 cells to aberrant AT2 cells (Fig. 3e, S4c). The epithelial clusters were largely negative for airway epithelial markers such as S100A2, KRT5, and CYP2F1 (S4a/b) (Sikkema et al. 2023). Trajectory analysis for both groups (with and without fibroblasts) showed that proliferating cells differentiated in two directions: AT2 and dedifferentiated cells (Fig. 3f). Together, these results indicate that fibroblasts drive AT2 cells towards MUC5B-expressing AT2-like cells in this model.

### Fibroblasts induce expression of MUC5B in pneumocytes

**Fig. 4:**
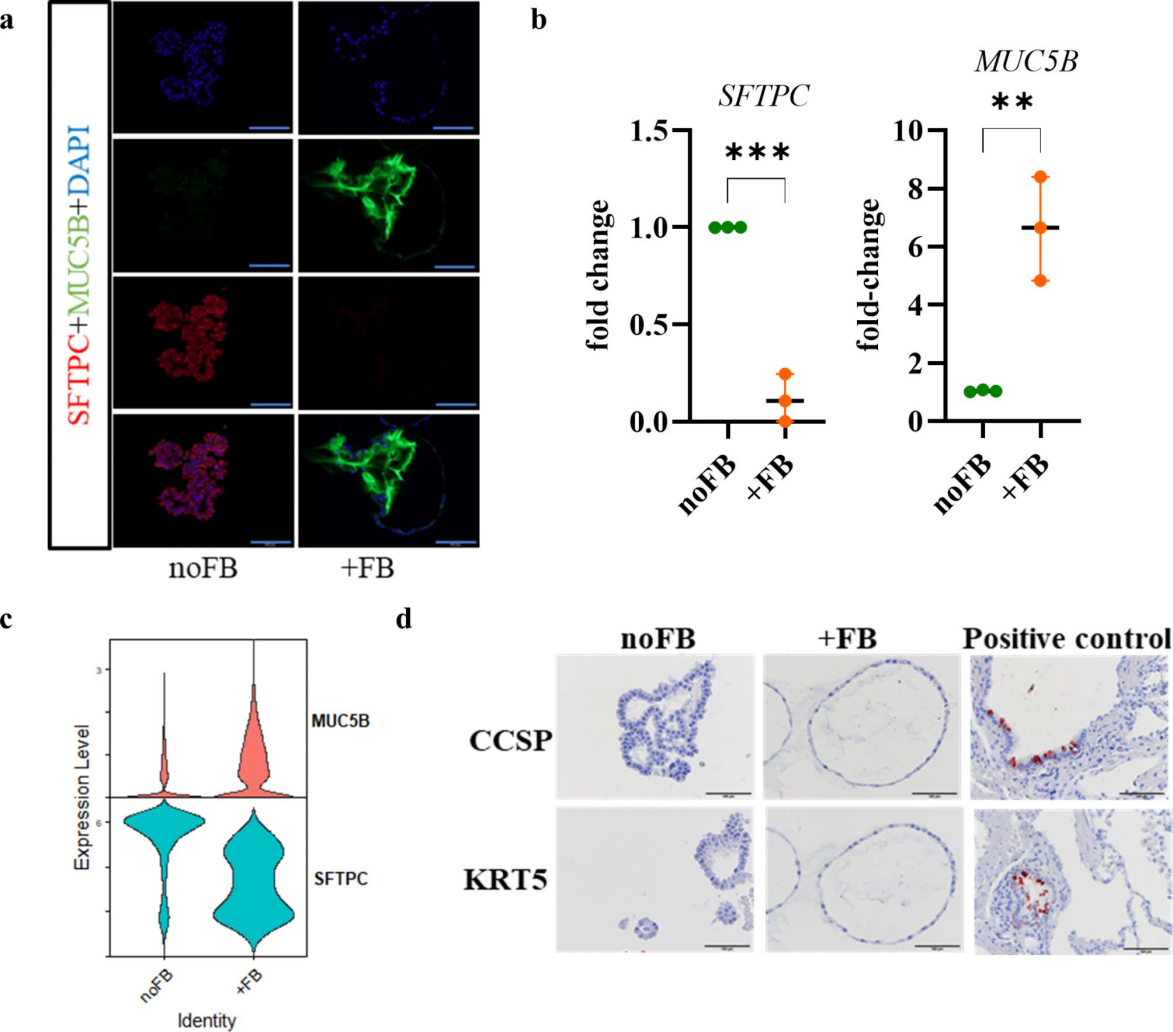
Fibroblasts induce the expression of MUC5B. (**a**) Organoids were stained for SFTPC and MUC5B by immunofluorescence (Scale bar = 100 μm). (**b**) The expression of SFTPC and MUC5B was confirmed by semi-quantitative RT-PCR. Data were compared by unpaired t test (**p < 0.01, ***p < 0.001). Each data point represents an independent experiment. (**c**) Violin plots showing expression of SFTPC and MUC5B. (**d**) Immunohistochemistry was performed for CCSP and KRT5 (positive control: human lung tissue; scale bar = 100 μm)

**Fig. 5:**
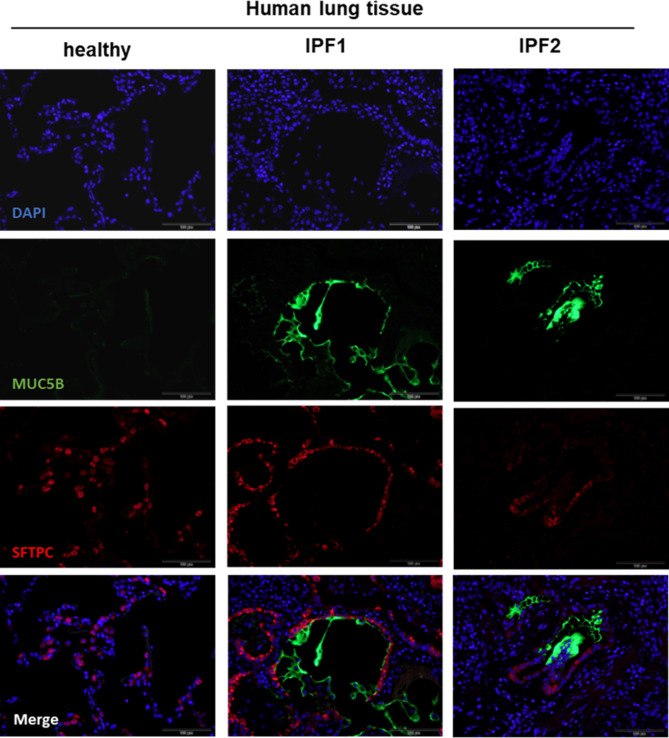
Strong expression of MUC5B in fibrotic lesions in lungs of IPF patients. MUC5B and SFTPC were detected by immunofluorescence in human lung samples obtained from a healthy donor and two IPF patients (scale bar = 100 μm)

Immunofluorescence microscopy and semi-quantitative RT-PCR analyzes showed, in addition to the single cell analyses, that the co-culture with fibroblasts from all three donors led to a reduced expression of SFTPC and a significantly increased expression of MUC5B (Fig. 4a to c, also Fig. 5d). Increased expression of MUC5B was not present in co-cultures with MRC5 cells (S2C). However, co-culture with MRC5 cells results in a decreased expression of SFTPC and thus in a loss of AT2 identity (S2C). Consistent with the single cell analysis, the organoids were negative for the airway epithelial markers KRT5 and SCGB1A1 (Fig. 4D).

Staining of lung tissue from IPF patients showed strong expression of MUC5B in fibrotic lesions partially associated with SFTPC-expressing cells (Fig. 5, S5). Re-analysis of the single-cell data published by Habermann et al. (Habermann et al. 2020) revealed around 25% of MUC5B-expressing cells in the AT2 cell cluster of IPF patients (S6).

### IL-6-STAT3 signaling drives MUC5B-expression in AT2 cells

**Fig. 6:**
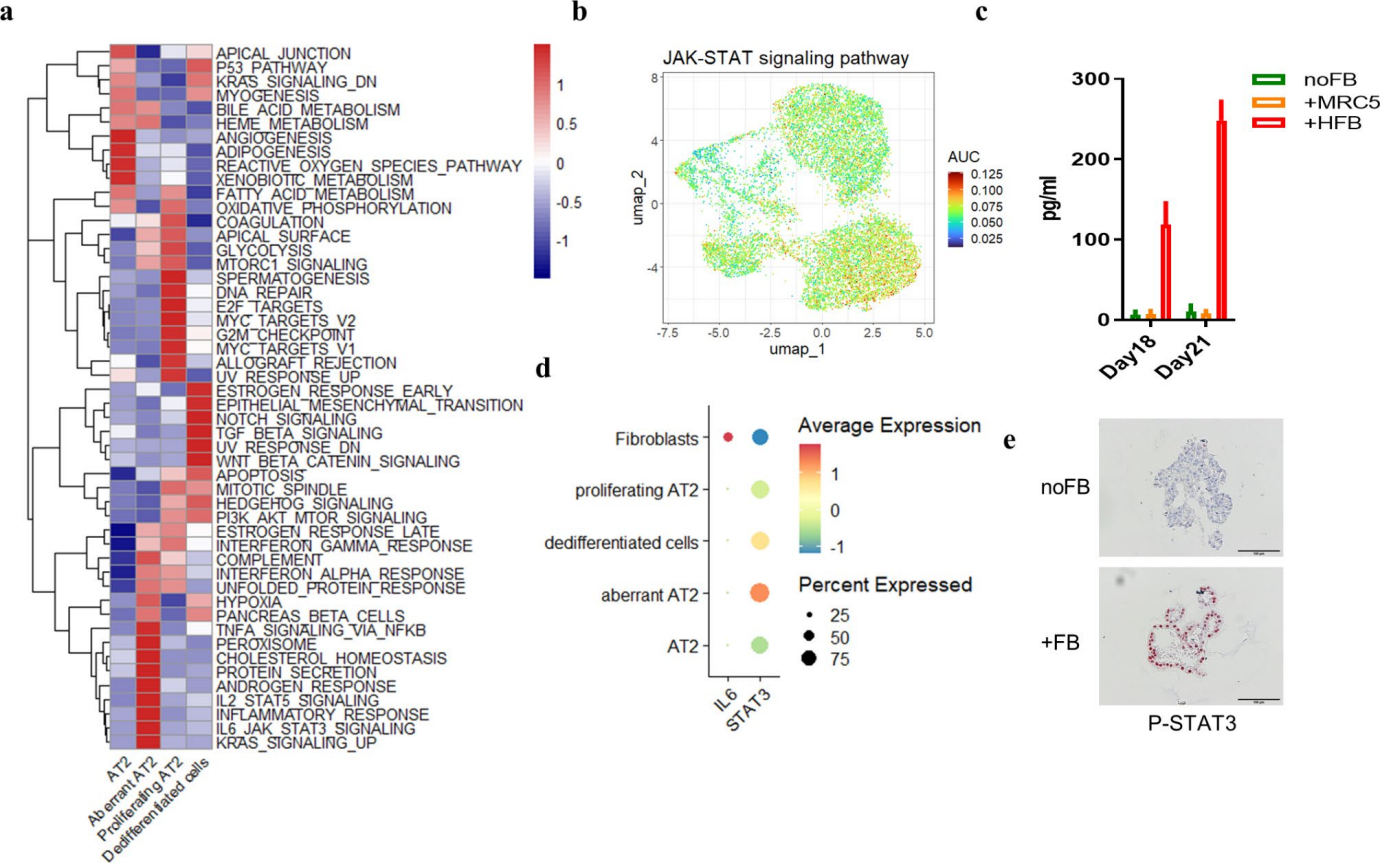
Fibroblasts activate IL-6/STAT3 pathways. Signaling pathways were analyzed by (**a**) GSVA and (**b**) AUCell. (**c**) IL-6 was measured in supernatants of the cultures at day 18 and 21. (**d**) Dot plot showing expression of IL-6 and STAT3. (**e**) Immunohistochemistry was performed for P-STAT3 (scale bar = 100 μm)

Gene set variation analysis (GSVA, Fig. 6a, S7a) and AUCell (Fig. 6b, S7b) showed that IL-6/STAT3 and TNF-α/NFκB pathways were activated in aberrant AT2 cells. The PI3K-Akt signaling pathway was strongly activated in fibroblasts (S7C). IL-6 concentrations were increased in supernatants from co-cultures with primary fibroblasts (Fig. 6c) and fibroblasts, but not epithelial cells expressed IL-6 (Fig. 6d). STAT3 was highly expressed and phosphorylated in nuclei of pneumocytes cultured with fibroblasts (Fig. 6d and e). Staining of lung tissue from IPF patients showed expression of IL-6 in fibrotic lesions partially associated with PGFRA-expressing cells (S8).

**Fig. 7:**
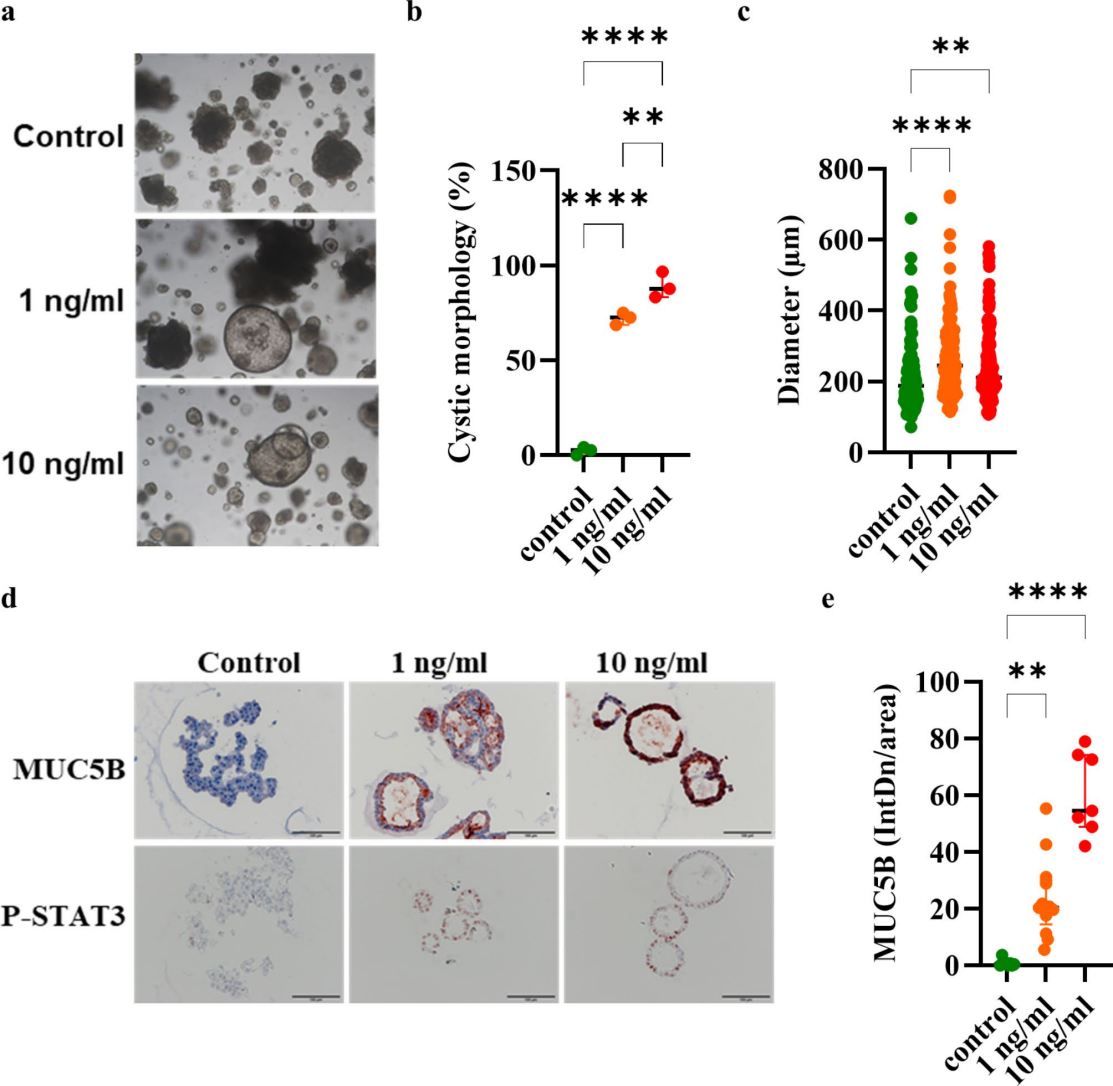
IL-6 induces the differentiation of AT2 cells towards a MUC5B^+^ cystic phenotype. The organoid cultures were stimulated with IL-6 from the day of seeding. (**a**) Representative phase contrast images of organoids cultured for 21 days. (**b**) Quantification of the morphology of the alveolar organoids. (**c**) Quantification of the diameter of the alveolar organoids. (**d**) Immunohistochemistry was performed for MUC5B and P-STAT3 (scale bar = 100 μm). (**e**) Quantification of MUC5B staining. Data were compared by one-way ANOVA (**p < 0.01, ****p < 0.0001)

As fibrotic fibroblasts express IL-6 and induce IL-6-STAT3 signaling (Fig. 6, S9), we examined, whether ongoing stimulation with IL-6 affects the phenotype of organoids. Stimulation with IL-6 from the day of seeding resulted in a cystic, MUC5B-expressing phenotype with nuclei positive for phosphorylated STAT3. IL-6 decreased the expression of SFTPC, but not NAPSA (Fig. 7, S10).

**Fig. 8:**
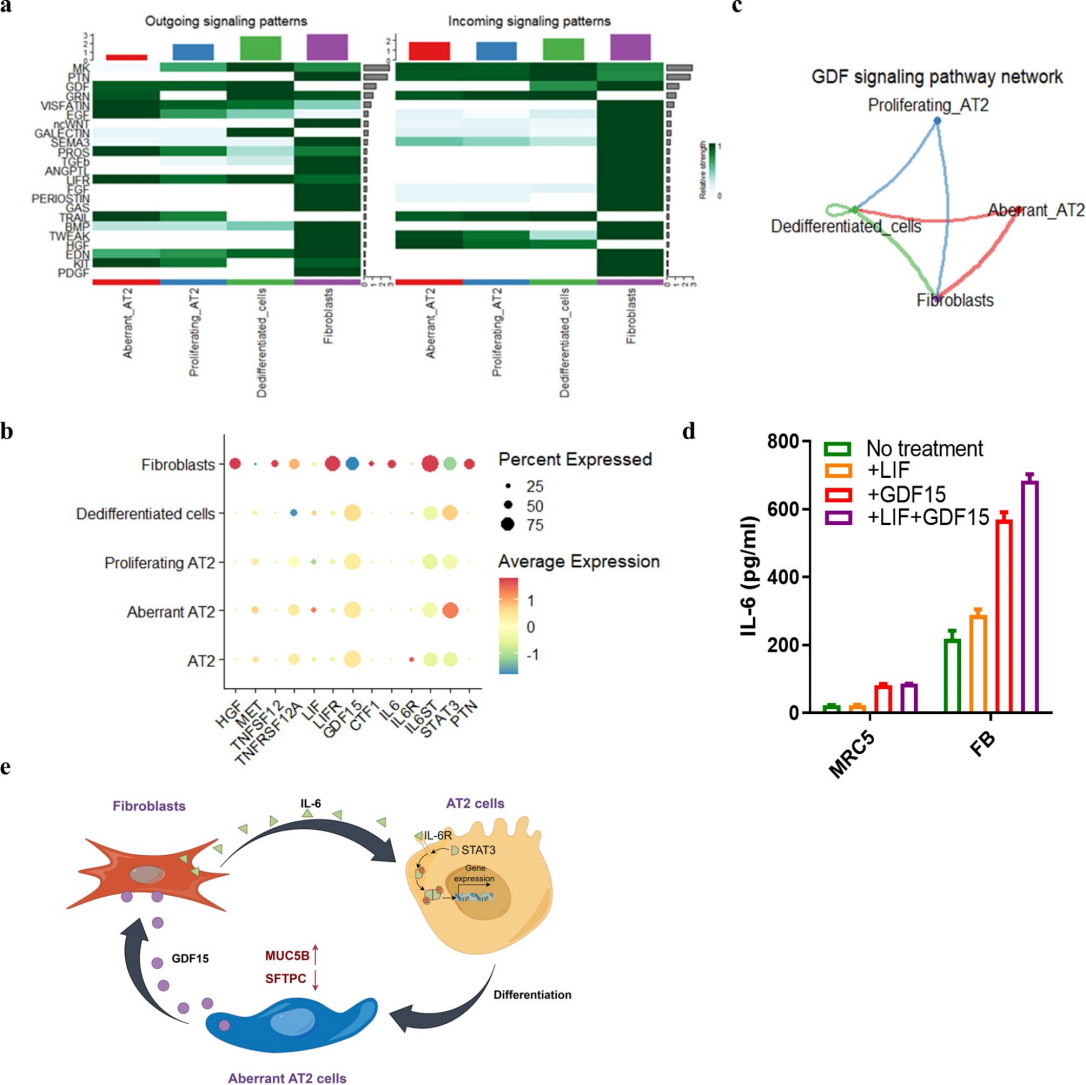
Fibroblasts activate IL-6/STAT3, TNF-α and HGF pathways in aberrant AT2 cells. (**a**) Heatmap (prediction of the CellChat algorithm) with outgoing and incoming signals in the different cell types. (**b**) Dot plot showing expression of selected ligands and receptors. (**c**) Signaling activity of GDF15 between epithelial cells and fibroblasts predicted by CellChat algorithm. (**d**) Primary fibroblasts and the cell line MRC5 were incubated for 24 h with GDF15 (200 ng/ml) and LIF (100 ng/ml) and IL-6 concentrations were measured in the supernatants. (**e**) Proposed schematic diagram of fibroblast-epithelial cell interaction.

In order to take a closer look at the interaction of the fibroblasts with the pneumocytes, ligand-receptor analysis was carried out for the pneumocyte-fibroblast co-cultures. Fibroblasts expressed HGF, TWEAK (TNFSF12) and PTN with predicted receptivity in the pneumocytes (Fig. 8a and b, S11a). Autocrine and paracrine induction of LIF/LIFR signaling, which has been shown to mediate IL-6 expression in fibroblasts (Nguyen et al. 2017), is predicted in fibroblasts (Fig. 8A, S11a and b). GDF15 expressed by epithelial cells (Fig. 8b, S11c) is predicted to signal from pneumocytes to fibroblasts (Fig. 8a and c). Primary fibroblasts, but not the cell line MRC5 expressed increased levels of IL-6 in response to GDF15 and LIF (Fig. 8d). Together, our data suggest that prolonged release of STAT3-activating factors by fibroblasts results in aberrant secretory AT2 cells that further increase IL-6 expression via epithelial factors such as GDF15 (Fig. 8E).

### Treatment with dasatinib reduces the formation of mucoid organoids

**Fig. 9:**
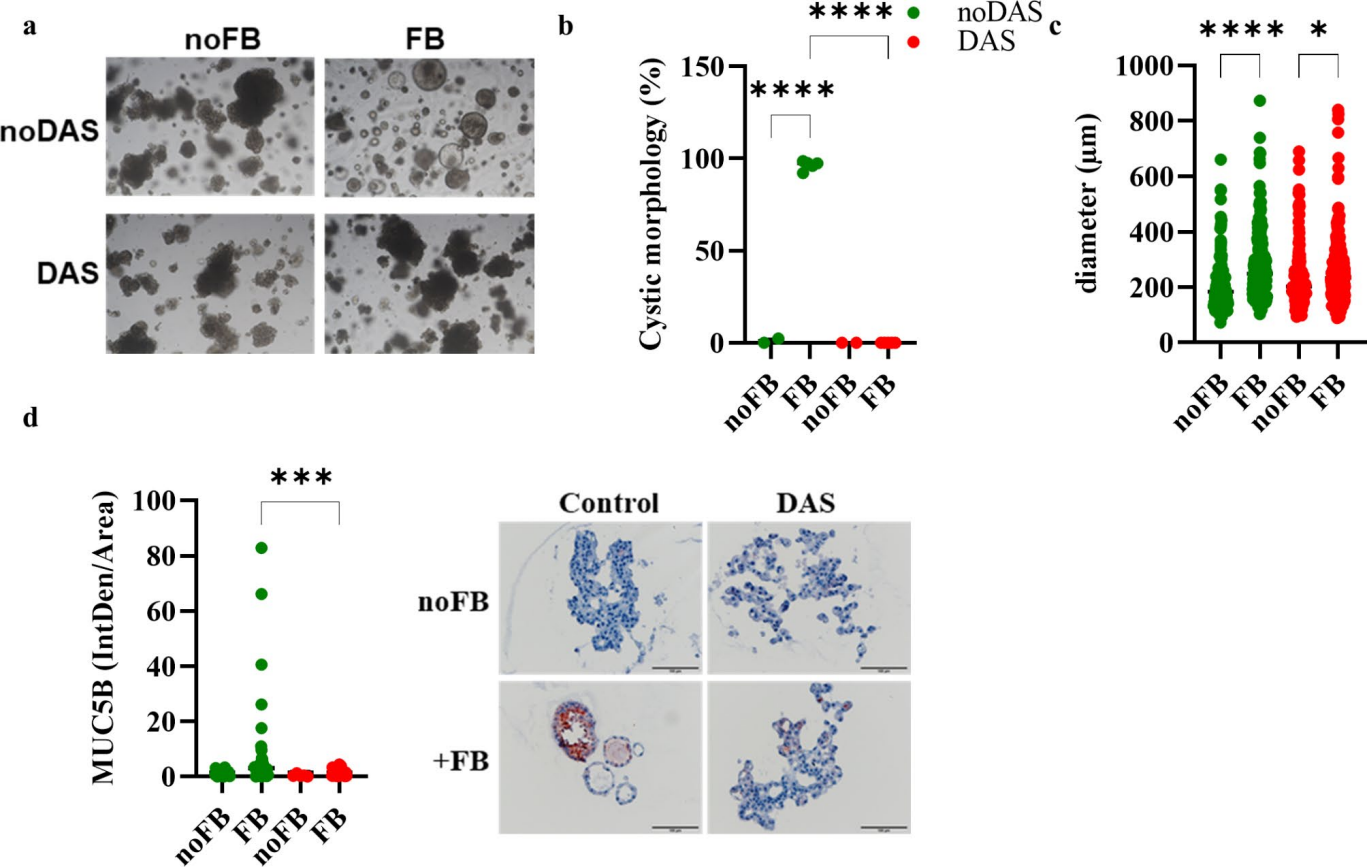
Dasatinib reduces cystic organoid formation. The organoid cultures were incubated with dasatinib (200 nM) from the day of seeding. (**A**) Representative phase contrast images of organoids cultured for 21 days. (**B**) Quantification of the morphology of the alveolar organoids. Each data point represents an independent experiment. (**C**) Quantification of the diameter of the alveolar organoids. (**D**) Immunohistochemistry was performed for MUC5B (scale bar = 100 μm). (**E**) Quantification of MUC5B staining. Pooled results from 3 independent experiments. Data were compared by one-way ANOVA (*p < 0.05, ***p < 0.01, ****p < 0.0001)

The single cell analyzes showed that the fibroblasts express markers for senescence and revealed activation for the PI3K-Akt signaling pathway (S7c, S12) (Saul et al. 2022; Moiseeva et al. 2023; Kohli et al. 2021). Using dasatinib (Zhu et al. 2015), we tested to what extent our model can be used to test drugs that counteract the secretory phenotype induced by fibrotic fibroblasts. For this purpose, we pretreated the fibroblasts of the three donors with dasatinib or control medium. Subsequently, co-cultures were established, with dasatinib still being added to the cultures with pretreated fibroblasts. In cultures without fibroblasts, dasatinib had no effect on grape-like morphology and organoid diameter. In contrast, treatment with dasatinib prevented the cystic growth of the organoids in the co-culture (Fig. 9a to c). In dasatinib-treated cultures, MUC5B expression was significantly reduced (9d, S13). However, dasatinib did not reverse the fibroblast-induced loss of SFTPC expression, but did reduce STAT3 phosphorylation (S13).

## Discussion

Here, we investigated the influence of primary fibroblasts on the differentiation of AT2 cells in our organoid model. We show that culturing AT2 cells in the presence of fibrotic fibroblasts results in secretory cystic organoids with reduced expression of SFTPC but increased expression of MUC5B. We identified regulatory circuits in which fibroblasts secrete inflammatory mediators such as IL-6 and activate pneumocytes, for example, via STAT3-dependent signaling pathways. We also demonstrate that the model is suitable for studying pharmacological interventions.

Our organoid model reveals high plasticity of AT2 cells, especially when cultured with mesenchymal cells. With a strong expression of SFTPB and SCGB3A2, low expression of SFTPC and no expression of SCGB1A1, the dedifferentiated cells resemble the recently described AT0 and terminal and respiratory bronchiole (TRB) secretory cells, which can differentiate from AT2 cells during lung regeneration (Murthy et al. 2022; Sikkema et al. 2023). In the presence of activated fibroblasts, the pneumocytes cells strongly lost the AT2 identity and developed a pronounced secretory phenotype.

A high plasticity of AT2 cells was also demonstrated by Kathiriya et al. in organoid models (Kathiriya et al. 2022). The authors showed that human AT2 cells, but not murine AT2 cells, transdifferentiate into basaloid cells when cultured in the presence of activated mesenchymal cells. Unlike the basaloid cells, the aberrant AT2 cells in our model did not show any expression of markers for airway epithelial cells such as KRT5 (Kathiriya et al. 2022; Adams et al. 2020). The differences between our study and Kathiriya et al. could be result from the different culture media used. The WNT-activator used in our study might prevent the differentiation of AT2 cells to KRT5^+^ basal cells. In our model there was also no evidence of intermediate steps, such as KRT17^+^/KRT8^High^ cells (S4) (Kathiriya et al. 2022), on the transdifferentiation pathway. Rather, there was a continuum from SFTPC-expressing to MUC5B-expressing cells. Future studies must show to what extent fibroblasts modulate the differentiation of AT2 cells towards a secretory phenotype in chronic lung diseases such as IPF, without further differentiation into basaloid cells.

The secretory aberrant AT2 cells in our model expressed MUC5B. MUC5B is typically produced by airway epithelial cells such as goblet cells and not by pneumocytes (Sikkema et al. 2023). Numerous preclinical and clinical studies indicate an important role of MUC5B in the pathogenesis of IPF. MUC5B promoter variants are a dominant risk factor for the development of IPF (Moore et al. 2019; Borie et al. 2013, 2022; Seibold et al. 2011; Fingerlin et al. 2013; Zhang et al. 2011; Stock et al. 2013; Peljto et al. 2015). Furthermore, MUC5B is abundantly expressed in honeycomb cysts, which are characteristic structures in fibrotic lesions, as well as in the distal airways of IPF patients (Conti et al. 2016; Hancock et al. 2018). We also could detect a strong MUC5B expression which partially associated with SFTPC^+^ cells. Hancock et al. showed that, in IPF patients, MUC5B is co-expressed with SFTPC in epithelial cells lining the honeycomb cyst and in AT2 cells, suggesting that epithelial cells in the lung parenchyma express MUC5B in IPF (Hancock et al. 2018). Forced expression of *Muc5B* under the control of the *Sftpc* promoter in the distal lung worsened the outcome in the bleomycin-induced mouse model (Hancock et al. 2018; Kurche et al. 2019). Although numerous cell types in the IPF lung likely contribute to deleterious expression of MUC5B, our results suggest that fibrotic fibroblasts drive AT2 cells toward MUC5B-expressing AT2 cells, as described for honeycomb cysts (Conti et al. 2016; Hancock et al. 2018).

Our single-cell data showed that the fibroblasts in the co-culture model adopted an IPF-like phenotype, similar to fibroblasts in IPF lungs and the mouse fibrosis model (Kathiriya et al. 2022; Peyser et al. 2019; Adams et al. 2020; Jia et al. 2023). The analysis also showed that fibroblasts activate signaling pathways (e.g. STAT3-, NFκB-, and HGF-dependent pathways) in pneumocytes that have a strong influence on cell differentiation. Conversely, pneumocytes had an influence on signaling pathways (e.g. LIF/LIFR signaling) related to the expression of IL-6 in fibroblasts (Nguyen et al. 2017). In vitro and mouse studies suggest that IL-6 released by fibroblasts promotes AT2 self-renewal and lung regeneration through STAT3-signaling (Zepp et al. 2017; Liang et al. 2016; Yao et al. 2023; Paris et al. 2020). However, IL-6-STAT3 signaling has been shown to play a central role in murine pulmonary fibrosis models (Le et al. 2014; O’Donoghue et al. 2012; Pedroza et al. 2016) and phosphorylated STAT3 was detected in nuclei of pneumocytes next to fibrotic lesions in the lungs of IPF patients (Pedroza et al. 2016). Thus, it is conceivable that an out-of-control activation of signaling pathways that are initially important for pneumocyte regeneration, such as IL6/STAT3-sighnaling, leads to a dysregulated pneumocytes with a secretory pneumocyte.

Our finding that profibrotic fibroblasts cause loss of AT-2 identity is also supported by further ex vivo studies showing that treatment of precision-cut lung slices (PCLS) and organoids with a fibrosis cocktail leads to reduced expression of AT-2 markers such as SFTPC (Alsafadi et al. 2017; Ptasinski et al. 2023; Lehmann et al. 2018). Kastlmeier showed that pluripotent stem cell-derived organoids lose AT2 identity when co-cultured with lung fibroblasts from fibrotic ILD patients (Kastlmeier et al. 2023). Bleomycin- and H_2_O_2_-treated human fibroblasts also reduced the progenitor potential of alveolar epithelial stem cells in the organoid model (Melo-Narvaez et al. 2024). Thus, organoid models depict aspects of disease and are therefore suitable for testing pharmacological compounds, especially in preclinical research before animal experiments (Brand et al. 2024).

Senescence is thought to play a role in the pathogenicity of chronic lung disease (Barnes et al. 2019; Hamsanathan et al. 2019). Our single cell analyzes showed strong expression of markers associated with senescence (e.g. CDKN2A, TIMP2, IL-6) and activation of the PI3K-Akt signaling pathway in fibroblasts. Thus, to test whether our model is suitable for pharmacological interventions, we treated the cultures with the drug dasatinib, which, among other things, is considered a senolytic agent that acts on the PI3K signaling pathway (Chaib et al. 2022). Dasatinib treatment resulted in increased expression of SFTPC and decreased expression of MUC5B in cultures lacking fibroblasts. Furthermore, dasatinib counteracted the cystic phenotype and fibroblast-induced expression of MUC5B without leading to an AT2 phenotype comparable to cultures without fibroblasts, at least with respect to the expression of SFTPC. Further studies are needed to elucidate the exact mechanism of action of agents such as dasatinib and other approved drugs (e.g. metformin) as well as natural compounds in relation to impaired pneumocyte differentiation and the formation of the recently discovered basaloid cells (Kathiriya et al. 2022; Adams et al. 2020; Jaeger et al. 2022). Moreover, the question arises to what extent the expression of MUC5B contributes significantly to pulmonary fibrosis and whether drugs that suppress the expression of MUC5B are helpful. Cell culture models, such as ours, can undoubtedly make a significant contribution to the testing of active ingredients with respect to their effects on diverse cell types and cellular mechanisms within the human system.

Based on the activation status of the included cells, our organoid model has limitations. Fibroblasts are highly activated in such models (Kathiriya et al. 2022; Melo-Narvaez et al. 2024), probably because they activate different pathways, such as repair programmes, in this unnatural environment. The required expansion of patient-derived fibroblasts for one to two weeks in conventional 2D culture also induces a fibrosis-like phenotype (Habermann et al. 2020). It is therefore almost impossible to introduce fibroblasts into 3D co-culture models as they exist in the donor lung, and difficult to study the extent to which fibroblasts from healthy and diseased tissue differ. However, the model allows direct assessment of pro-fibrotic activity without the need for exogenous application of additional fibrosis-inducing factors such as a fibrosis cocktail (Alsafadi et al. 2017; Ptasinski et al. 2023; Lehmann et al. 2018). Moreover, the medium contains several components (e.g., the WNT activator CHIR99021) that can affect fibroblast behavior in a complex manner, such as the release of growth and fibrotic factors. Due to donor variability, independent experiments with cells from different donors should always be performed in complex models using primary cells for drug testing. The complex isolation and cultivation of primary organoids is a significant additional effort in comparison to cell line models and conventional 2D cultures.

In summary, our results show that activated fibroblast induce a secretory phenotype characterized by MUC5B expression in AT2 organoids. Remarkably, this was not accompanied by the expression of respiratory epithelial markers. Excessive fibroblast-induced activation of signaling pathways associated with epithelial regeneration leads to a pathogenic AT2 phenotype in our model, as might also be the case in chronic lung diseases such as IPF. The model is well suited for testing active ingredients, e.g. in pre-clinical research.

## Materials and methods

### Sex as a biological variable

Sex was not considered as a biological variable.

### Human lung alveolar epithelial cell isolation and sorting

Alveolar epithelial cells were isolated from surgically removed lung tissue from the patient. The protocol for human material has been approved by the Landesärztekammer des Saarlandes Ethics Committee and informed consent has been obtained from all patients. The tissue was cut into small pieces and digested with a digestion solution containing 2.5 mg/ml collagenase type I (Life technologies, 17100-017), 1 ml dispase (Corning, 354235), 1 mg/ml DNase1 (Roche, 10104159001) for 35 min at 37 °C. The obtained cell suspension was filtered through a 70 μm cell strainer and centrifuged at 450 x g for 10 min at 4 °C. The red blood cells were then lysed with ACK buffer (Gibco, A1049201), and the remaining cells were washed twice with PBS containing 1% FBS (Gibco, 10270106), 1 mM EDTA (Roth, 8043.2). Then, HT2-280^+^ cells were obtained by incubating with HT2-280 antibody (Terrance, TB-27AHT2-280) for 1 h and afterwards with anti-mouse IgM beads (Miltenyi, 130-047-302) for 30 min in the dark at 4 °C. Labeled cells were sorted by MACS column (Miltenyi).

### Human lung fibroblasts isolation and culture

Lung (~ 1 cm^3^) tissue was cut into 6–8 individual pieces and cultured in petri dishes in DMEM medium (Gibco, 41965039) containing 10% FBS and 1% penicillin/streptomycin (Gibco, 1514). Media were changed every 4 days. Once the fibroblasts reached 80–90% confluence, cells were stored at -80 °C for future use.

### Human AT2 organoid culture and assay

AT2 organoid cultures were cultivated as described previously (Katsura et al. 2020). Briefly, HT2-280^+^ cells were resuspended in GFR-Matrigel and 5 × 10^3^ cells were seeded in each well of a 24-well plate. After 30 min of incubation at 37 °C to solidify the Matrigel, cells were cultured for 21 days with 500 µL of Advanced DMEM/F12 medium (Thermo Fisher Scientific, 12634010) containing 10 µM SB431542 (Abcam, Ab120163), 3 µM CHIR99021 (Tocris, 4423), 1 µM BIRB796 (Tocris, 5989), 50 ng/mL human EGF (Gibco, PHG0313), 10 ng/mL human FGF10 (BioLegend, 559304), 5 µg/mL heparin (Sigma-Aldrich, H3149), 1x B-27 supplement (Thermo Fisher Scientific, 17504044), 1x antibiotic-antimycotic (Gibco, 15240062), 15 mM HEPES (Thermo Fisher Scientific, 15630080), 1x GlutaMAX (Thermo Fisher Scientific, 35050061), and 1.25 mM N-acetyl-L-cysteine (Sigma-Aldrich, A9165). 10 µM of Y-27,632 (Sigma-Aldrich, Y0503) was added on the first 3 days of culture. The medium was changed every 3 days. For passaging, Matrigel was disrupted by incubation with dispase at 37 °C for 45 min, followed by single cell dissociation through the addition of TrypLE™ Express Enzyme (Gibco, 12605010) for 5 min at 37 °C. The cells were centrifuged at 450 x g for 5 min and resuspended in fresh GFR-Matrigel as before. In co-culture experiments, HT2-280^+^ and human lung fibroblasts were cultured in GFR-Matrigel at a 1:1 ratio for 21 days. In the IL-6 (PeproTech, 200-06-20UG) and Dasatinib (BMS-354825) experiments, 1 ng/ml or 10 ng/ml of IL-6 and 200 nM of Dasatinib were added to the medium for 21 days, respectively.

### Immunofluorescence (IF) and immunohistochemistry (IHC) staining

Organoids were embedded with 3% agarose as described before (Brand et al. 2024; Sprott et al. 2020). Primary antibodies for pro-SFTPC (Abcam, ab90716, 1/100) and MUC5B (Santa Cruz Biotechnology, sc-393952, 1/100) were used in IF staining. Primary antibodies for pro-SFTPC (1/5000, ab90716), MUC5B (1/500, sc-393952), KRT5 (1/1000, ab75869), NAPSA (1/1000, ab133249), CCSP (1/2000, ab307666) and STAT3 (1/500, 9145 S) were used in IHC staining. MUC5B staining intensity was quantified using ImageJ (National Institutes of Health, Bethesda, MD, USA) software and related to the area occupied by the organoid.

### qRT-PCR

Gene expression levels were quantified by qRT-PCR on the CFX 96TM Real-Time PCR Detection System (Bio-Rad) as described previously (Yao et al. 2024). Primers are listed in Table 1 in the online supplement.

### Single cell sequencing analysis

Organoids were digested into single cells using the methods described for passaging. Single cell analysis was performed using the BD Rhapsody™ Single-Cell Analysis System (Becton Dickinson, San Jose, CA, USA) according to manufacturer’s protocols. Samples were individually labelled using the Human Single-Cell Multiplexing Kit (BD, Cat. 633781) and subsequently pooled. Cells were captured, cell-specific mRNAs were transferred to barcoded capture beads, libraries were generated by use of the BD Rhapsody™ WTA Amplification Kit (BD, Cat. 633801) and finally sequenced on the Novaseq 6000 platform (Illimina, USA) with about 50,000 reads per cell. Raw sequencing reads were processed with the BD Rhapsody™ WTA Analysis Pipeline on the Sevenbridges cloud platform.

Data were processed with the Seurat package (version 5.0.1) in R software (version 4.3.1). Low-quality cells with gene expression < 2000 or > 10,000 genes or the percent of mitochondrial reads over 15% of total reads per cells were filtered out. The filtered dataset was normalized and scaled by using Seurat NormalizeData (scale factor 10,000) and ScaleData function with default parameters. Cell clusters were identified using a shared nearest neighbors (SNN)-based algorithm (resolution was set to 0.3). Nonlinear dimensional reduction was performend to generate UMAP plots as illustrated. GSVA (1.50.0) and AUCell (1.24.0) package in R was used for pathway activities analysis. The gene sets for signaling pathway activities were derived from “KEGG_2021_Human” list. CellChat (1.6.1) was used with default parameters for ligand-receptor interaction analysis. Single-cell pseudotime trajectories were generated with the Monocle3 package (Version 1.3.4) in R (Qiu et al. 2017).

## Electronic supplementary material

Below is the link to the electronic supplementary material.

Supplementary Material 1

## Data Availability

The single cell data is available at: https://0-www-ncbi-nlm-nih-gov.brum.beds.ac.uk/geo/query/acc.cgi?acc=GSE254441.

## Declarations

### Statistics

Graph Prism software (GraphPad Software (version 8.0), San Diego, CA) was used for statistical analysis. Comparisons between groups were analyzed by one-way ANOVA, unpaired t-test or Mann-Whitney test as indicated in the figure legends. The results were considered statistically significant for p < 0.05.
