# Supplementary material for: Co-culture of human AT2 cells with fibroblasts reveals a MUC5B phenotype: insights from an organoid model
Source: Mol Med. 2024 Nov 23;30:227. doi: 10.1186/s10020-024-00990-w (PMC11585087; doi:10.1186/s10020-024-00990-w)
Supplement: Supplementary file 1 — Supplementary Material 1 [file 10020_2024_990_MOESM1_ESM.pdf]

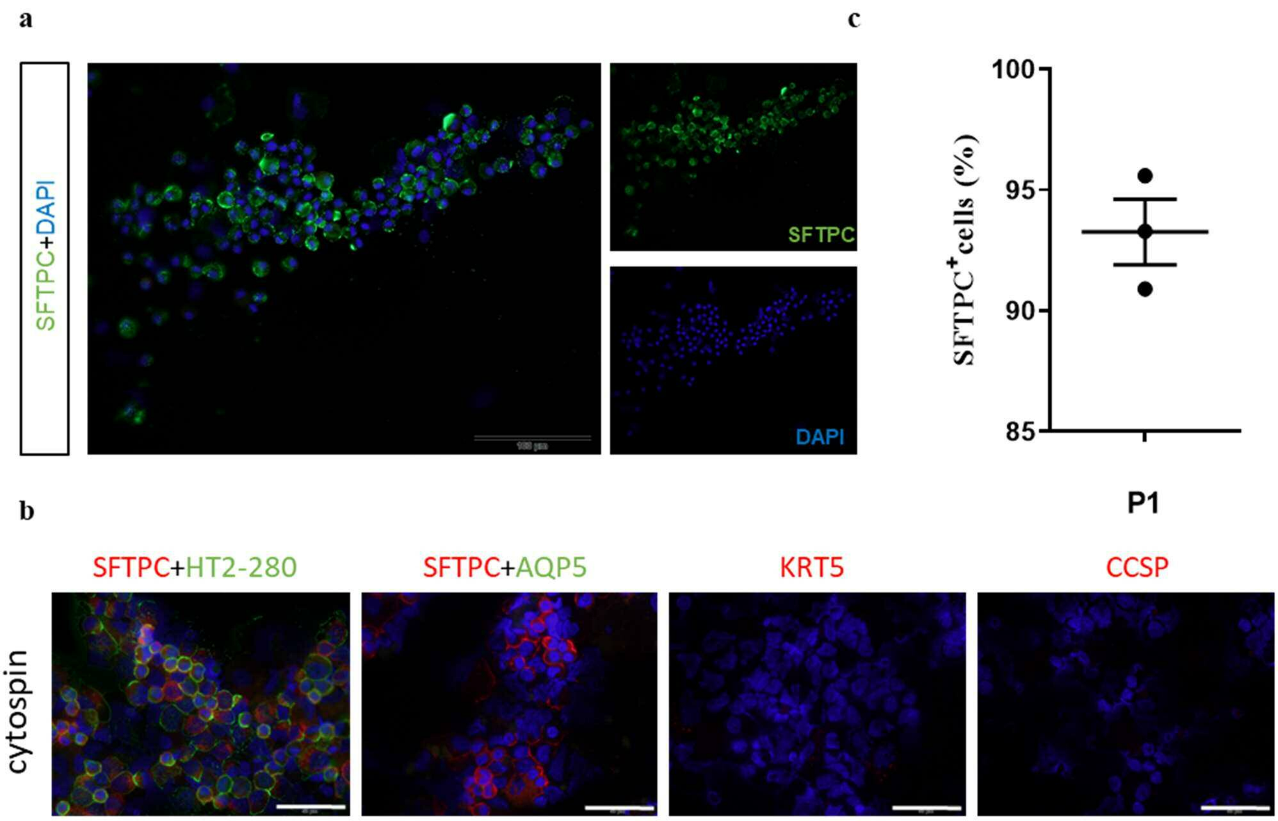

S1. Validation of AT2 cells. (a and b) Cytospins obtained immediately after passage (P1) were stained for SFTPC, HT2-280, AQP5, KRT5, and CCSP by immunofluorescence (Scale bar = 100 µm). (c) Percentage of cells positive for SFTPC was quantified (n=3 independent experiments, data are shown as mean ± SEM).

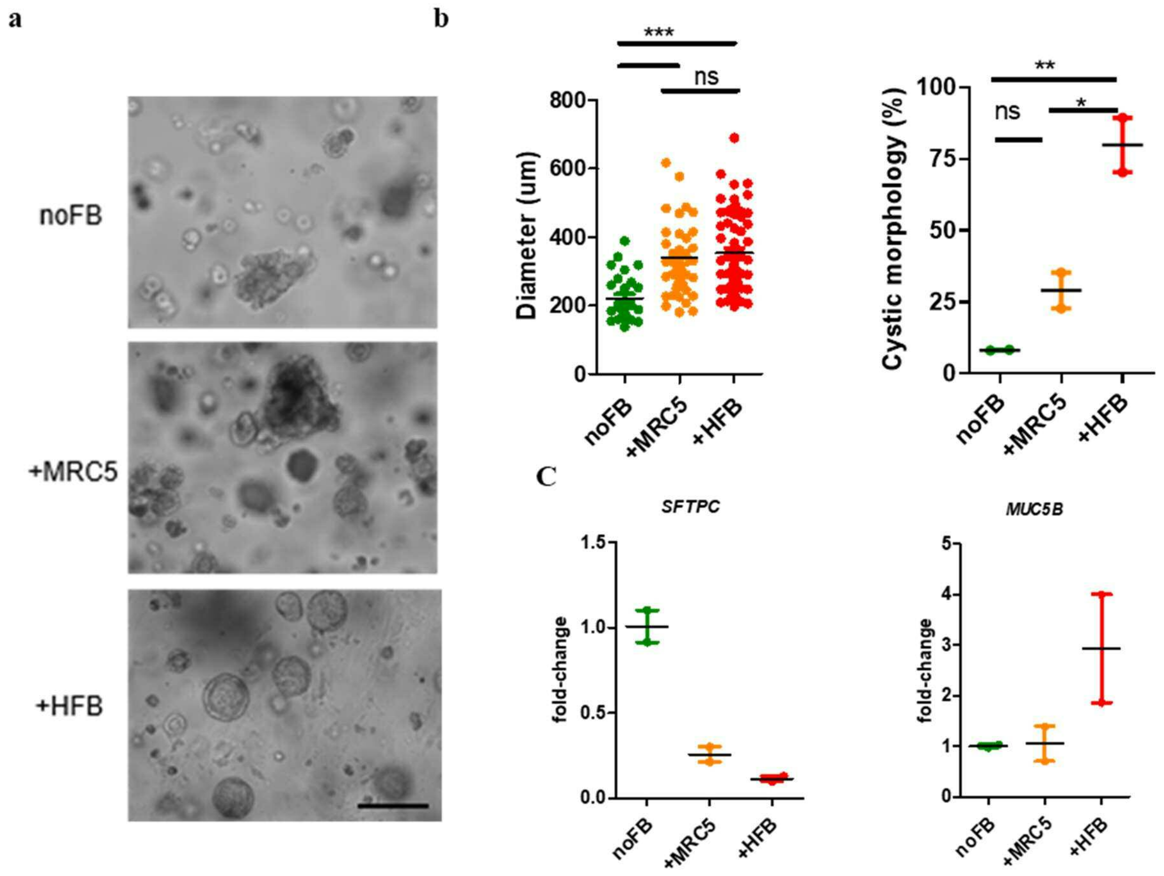

S2. (a) Representative phase contrast images of organoids cultured for 21 days (noFB: AT2 cells cultured without fibroblasts (FB); +HFB: AT2 cells cultured in the presence of fibroblasts from patients without chronic lung disease; +MRC5: AT2 cells cultured in the presence of the fibroblast cell line MRC5). (b) Quantification of the morphology and diameter of the alveolar organoids. (c) The expression of SFTPC and MUC5B was measured by semi-quantitative RT-PCR. Data were compared by one-way ANOVA. *p <0.05, **p < 0.01, ***p < 0.001.

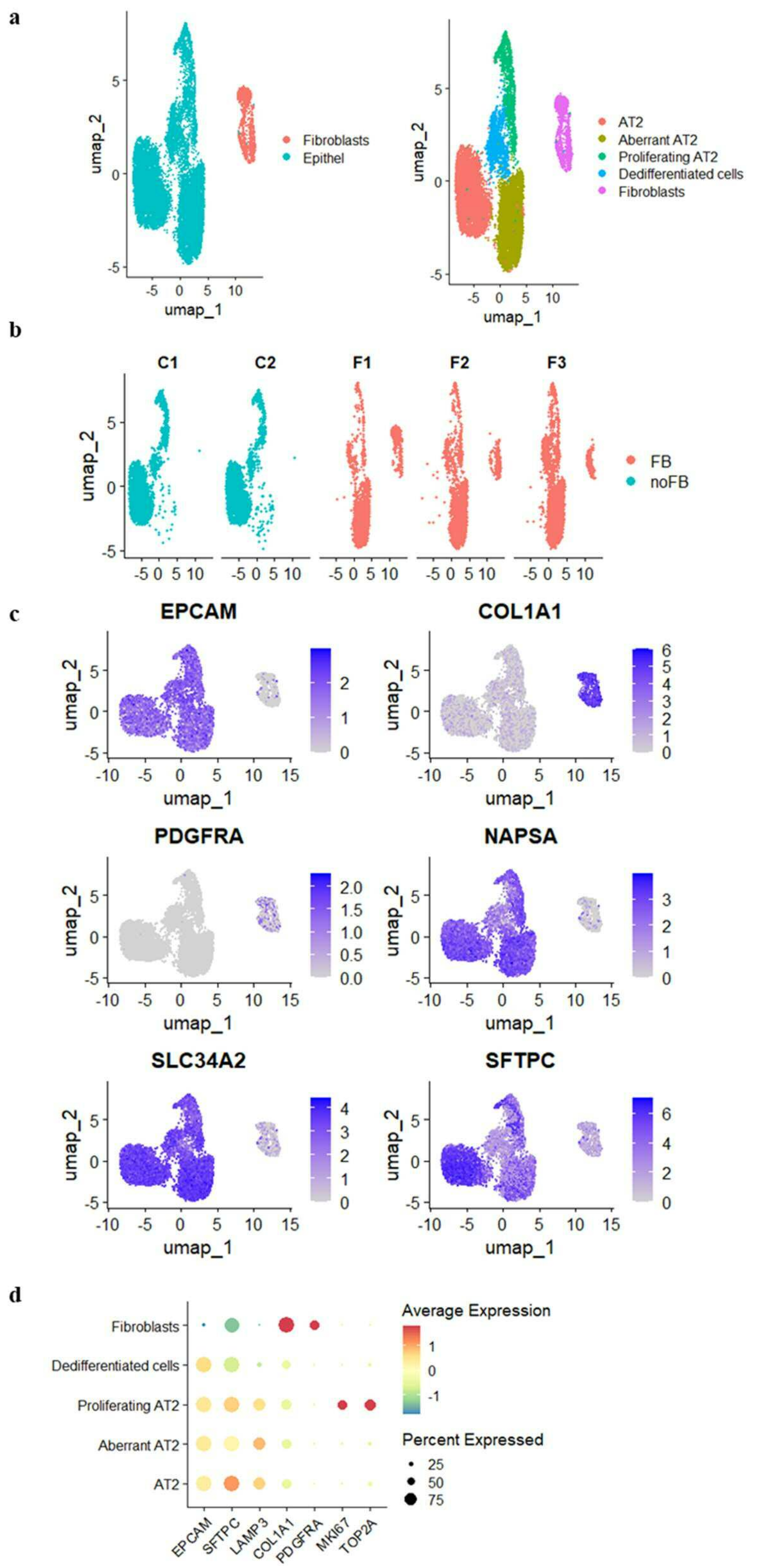

S3. scRNA-seq analysis of the organoids. (a) UMAP-visualization colored by cell type (red: fibroblasts. blue: epithelial cells). (b) UMAP visualization of the five groups (C1 and C2: control groups. F1, F2 and F3: co-cultures with fibroblasts from 3 different donors). (c) Feature plots of cell markers (epithelial cells and fibroblasts). (D) Dot plot showing expression of epithelial, fibroblast and proliferation markers.

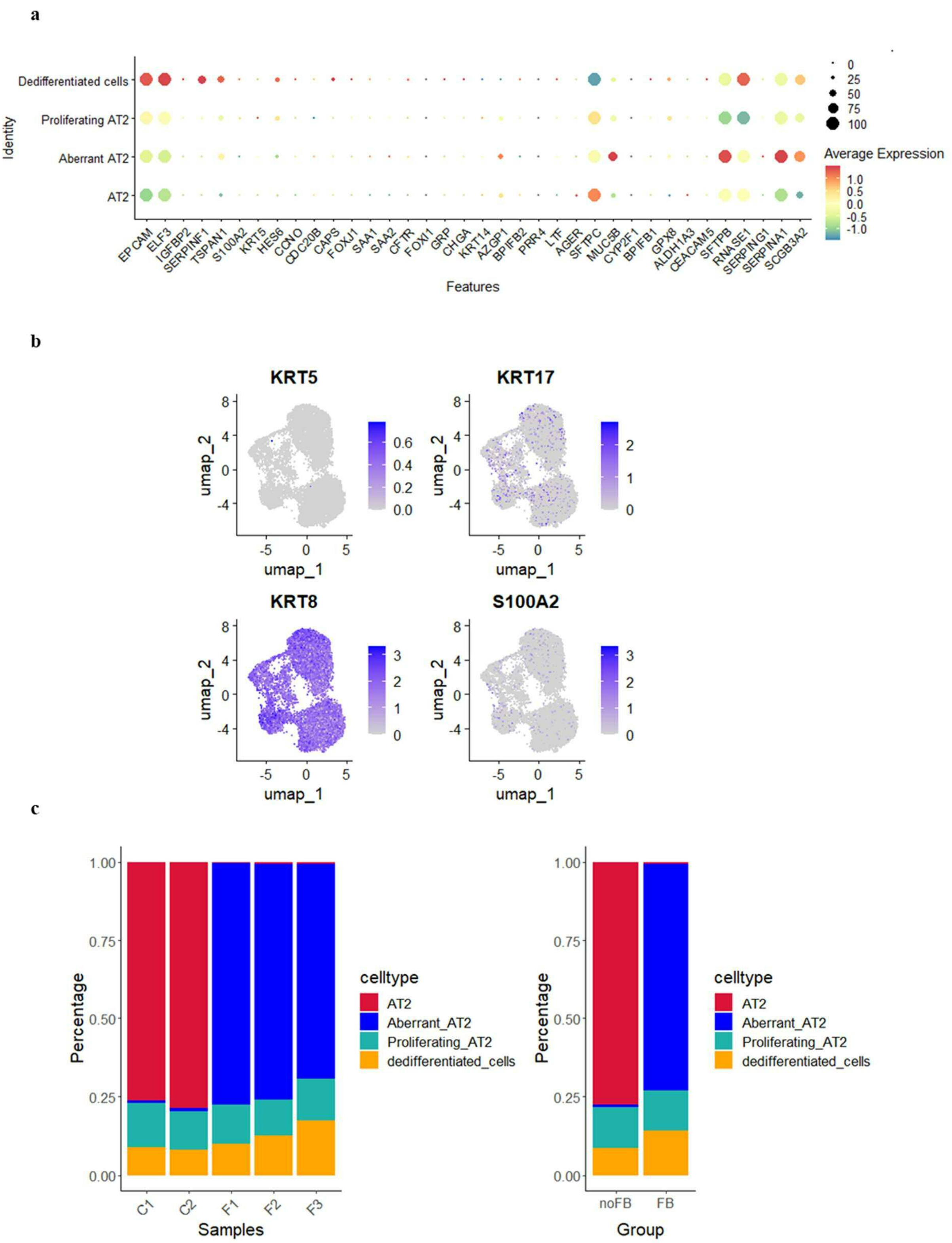

S4. (a) Dot plot showing expression of airway epithelial markers. (b) Feature plots of epithelial markers (c). Proportion of the different cell clusters by samples and groups (noFB: AT2 cells cultured without fibroblasts; FB: AT2 cells cultured in the presence of fibroblasts).

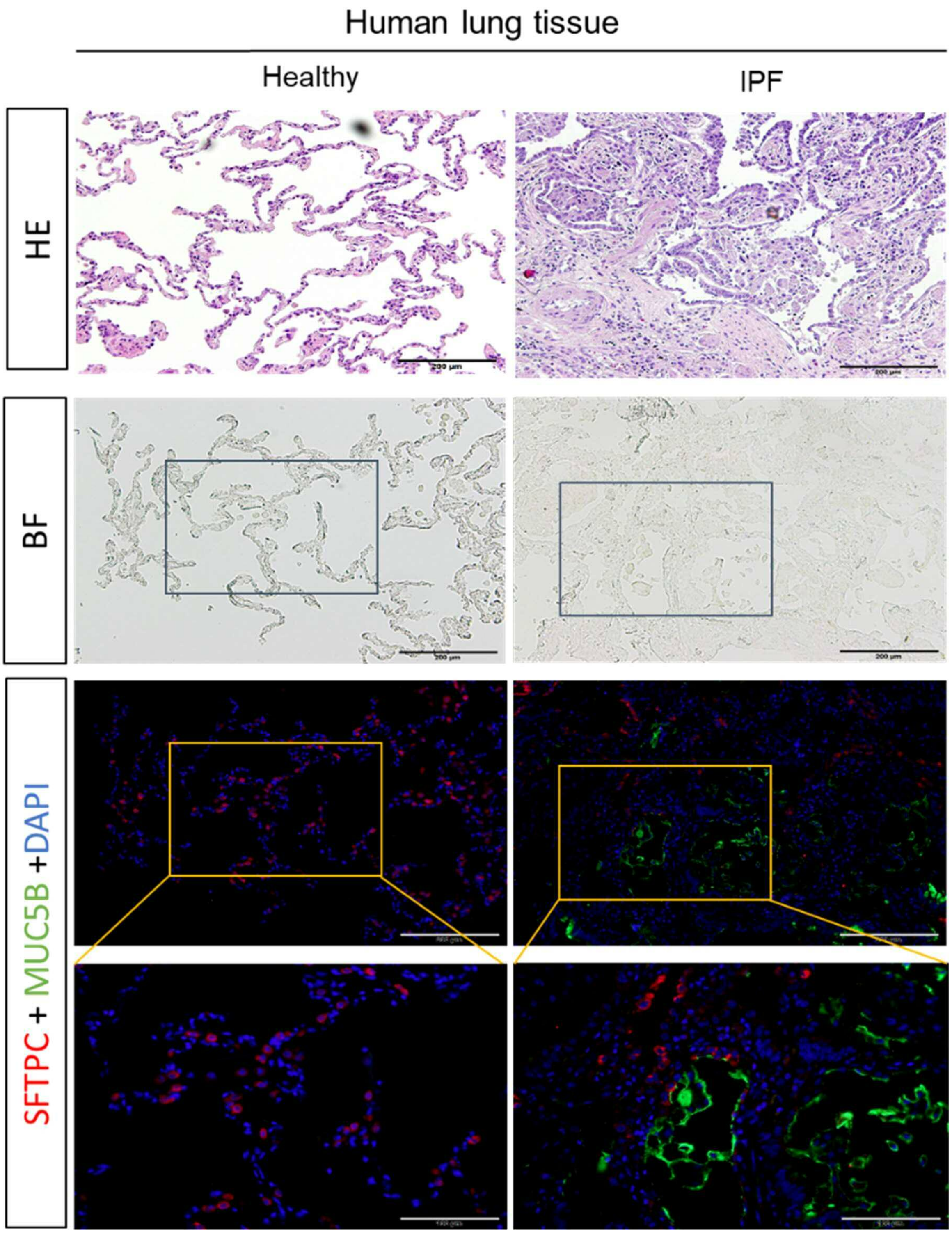

S5. Expression of MUC5B in fibrotic lesion in lungs of IPF patients. MUC5B and SFTPC were detected by immunofluorescence in human lung samples obtained from a healthy donor and an IPF patients (scale bar = 200 and 100 μm (below)).

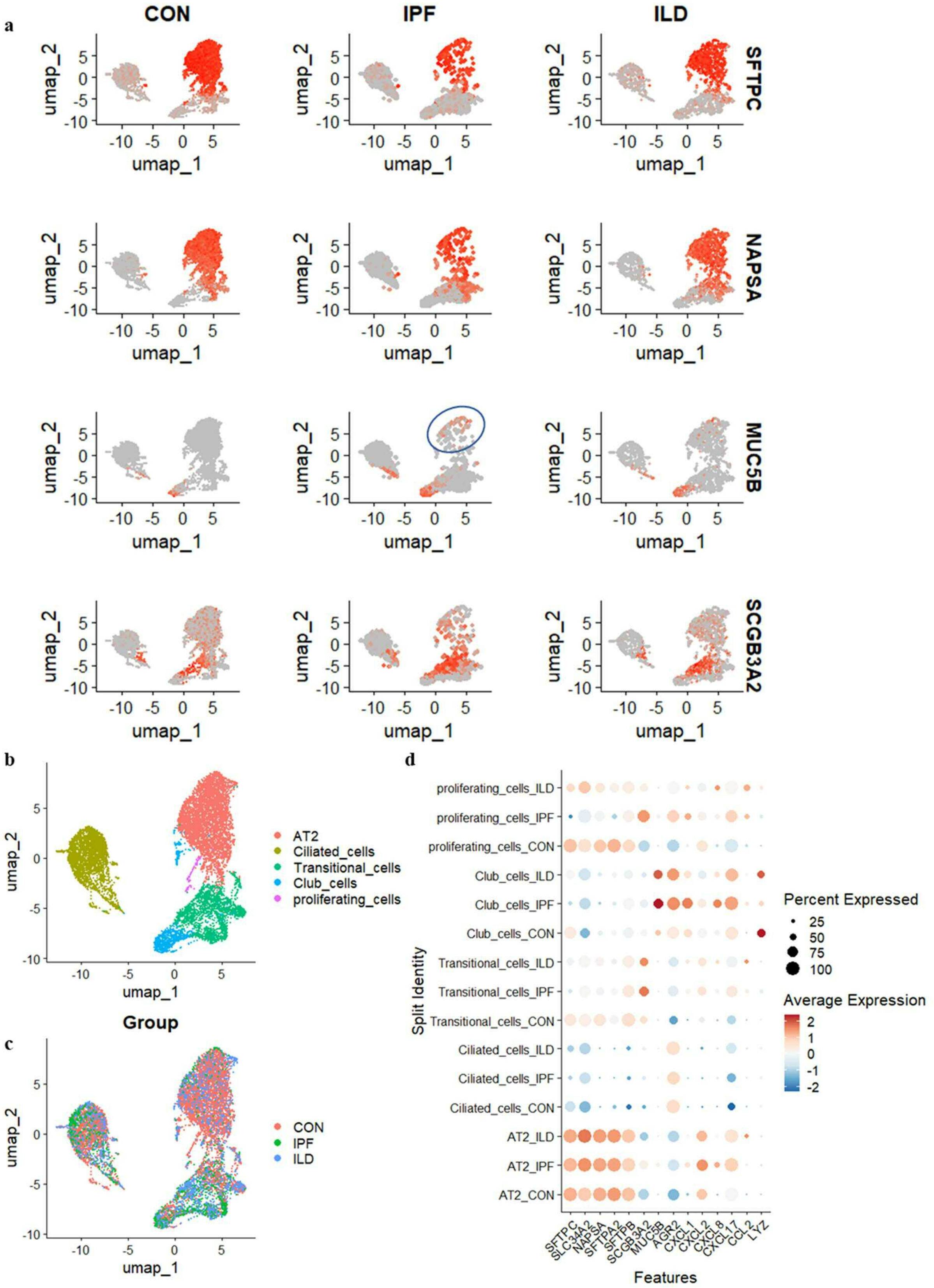

S6. RNA-seq reanalysis of human single cell data (1). (a) Feature plots showing the expression of selected genes in healthy donors (CON), patients with idiopathic lung fibrosis (IPF) and patients with interstitial lung diseases (ILD). (B) UMAP visualization of epithelial cells colored by (b) cell types and (c) groups. (d) Dot plot showing expression of epithelial markers and secretory factors marker

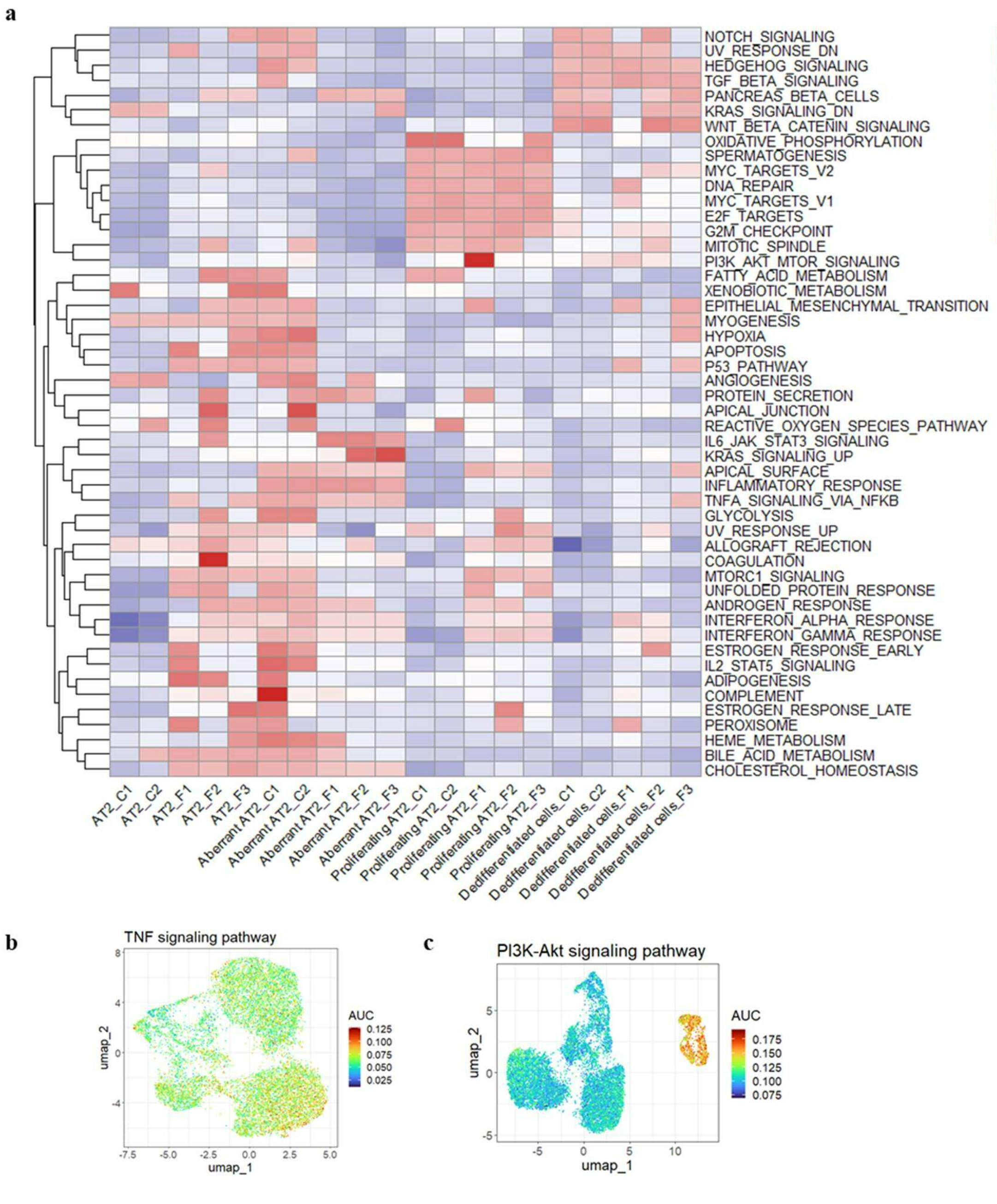

S7. Signaling pathways were analyzed by (a) GSVA and (b/c) AUCell.

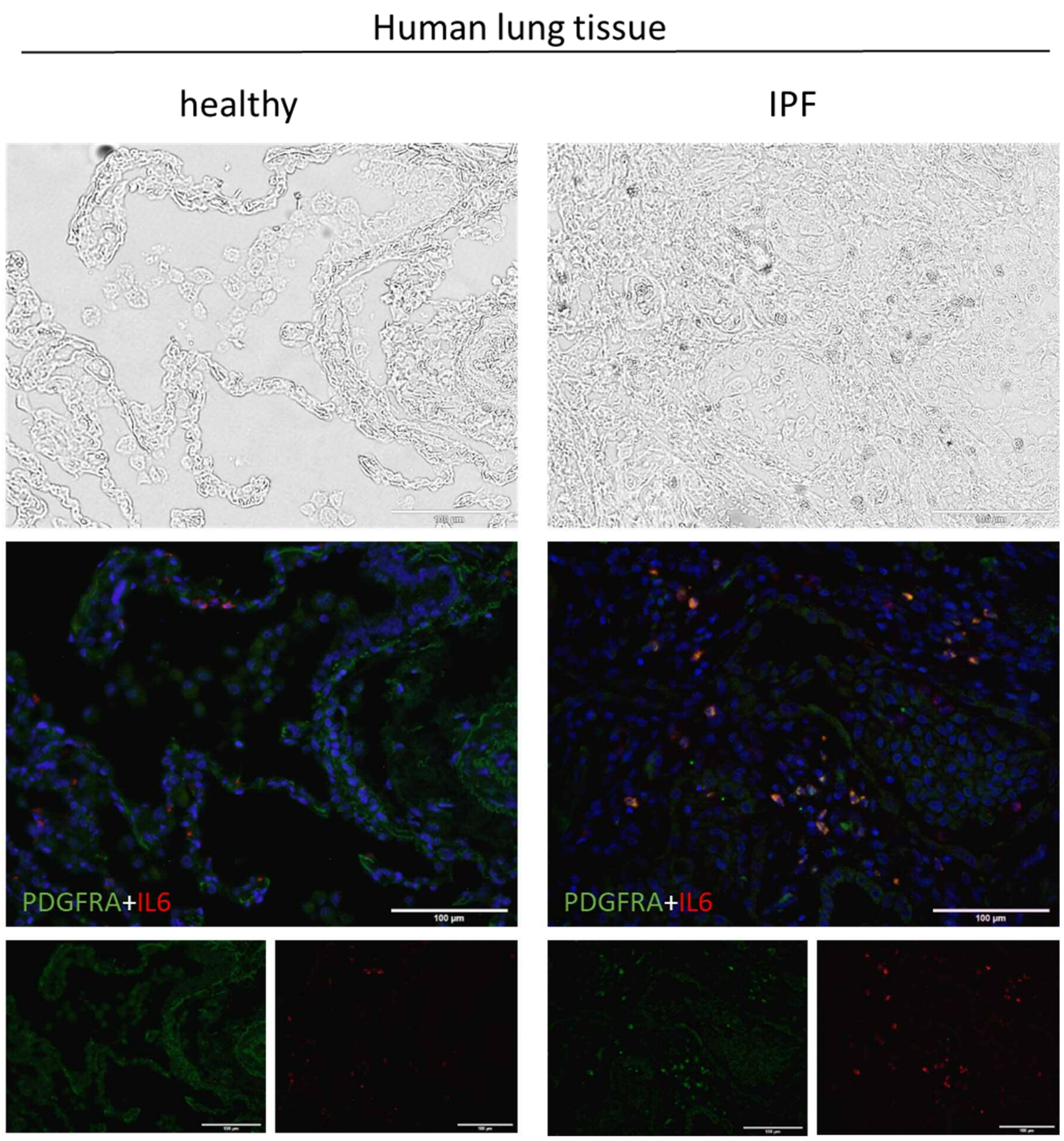

S8. Expression of IL6 in fibrotic lesion in lungs of IPF patients. IL6 and PDGFRA were detected by immunofluorescence in human lung samples obtained from a healthy donor and an IPF patients (scale bar = 100 µm).

a

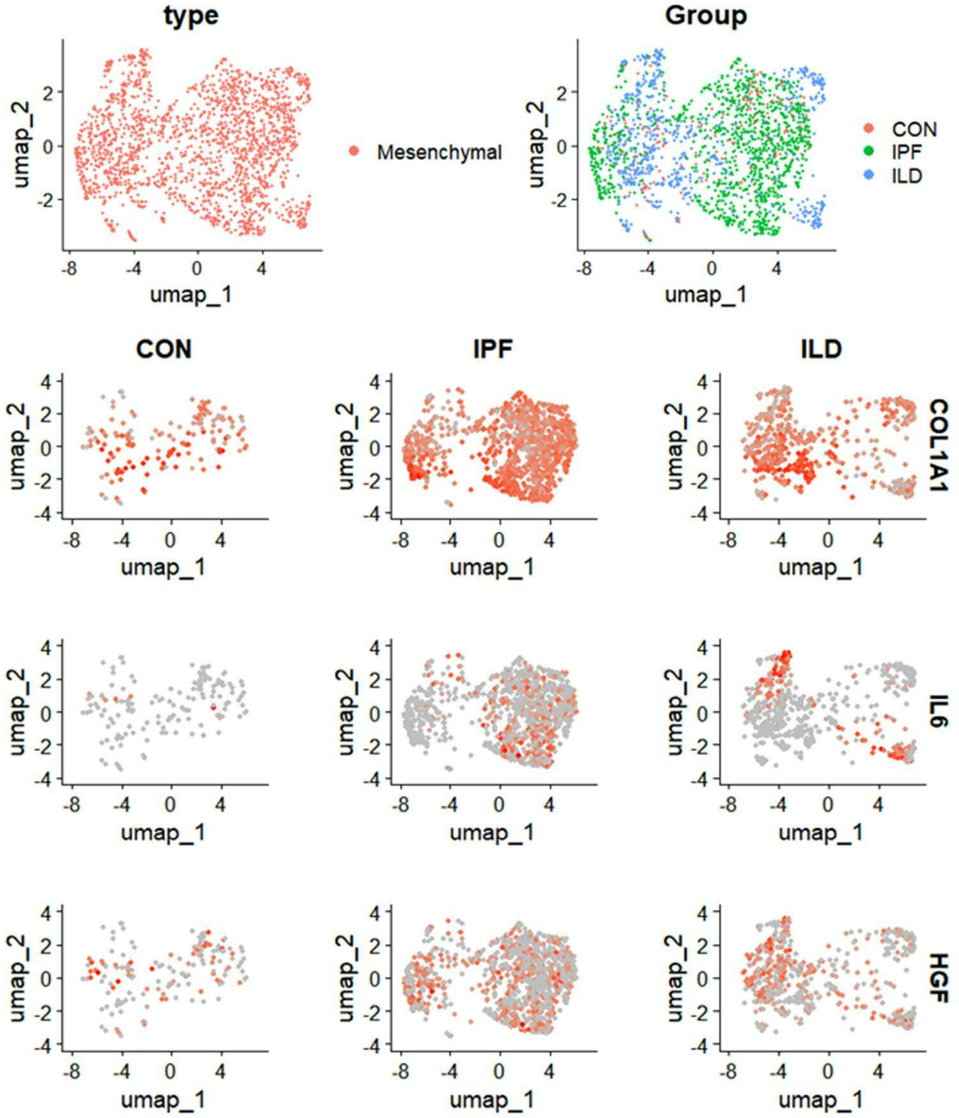

S9. RNA-seq reanalysis of human single cell data (1). (a) UMAP visualization colored by groups. (b) Feature plots showing the expression of selected genes in healthy donors (CON), patients with idiopathic lung fibrosis (IPF) and patients with interstitial lung diseases (ILD).

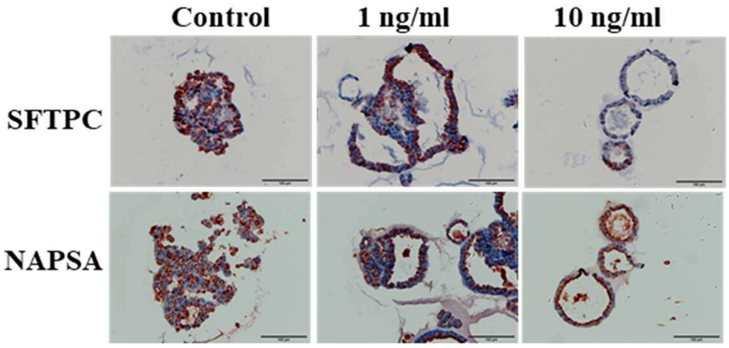

S10. The organoid cultures were stimulated with IL-6 from the day of seeding. Immunohistochemistry was performed for SFTPC and NAPSA (scale bar = 100 μm).

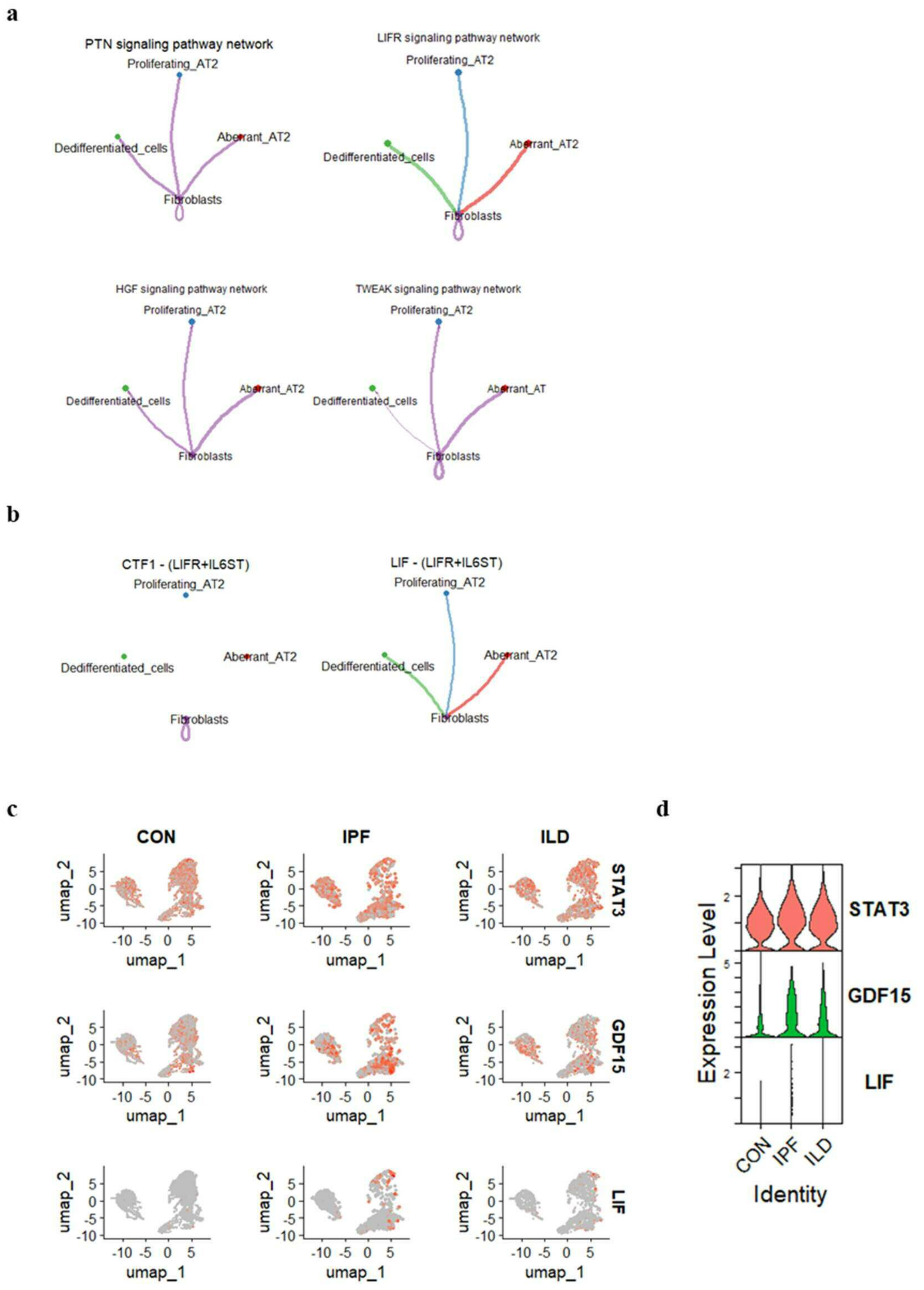

S11. (a) ligand-receptor interaction between epithelial cells and fibroblasts and (b) Signaling activity of selected pathways predicted the CellChat algorithm. (c and d) RNA-seq reanalysis of human single cell data. (c) Feature plots showing the expression of selected genes in healthy donors (CON), patients with idiopathic lung fibrosis (IPF) and patients with interstitial lung diseases (ILD). (d) Violin plot visualization of the markers.

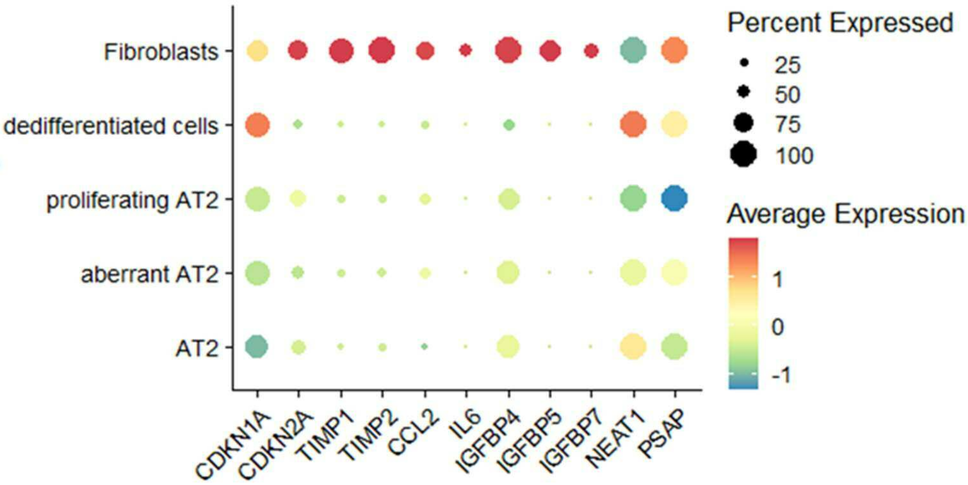

S12. Intensity dot plot showing expression of markers for senescence.

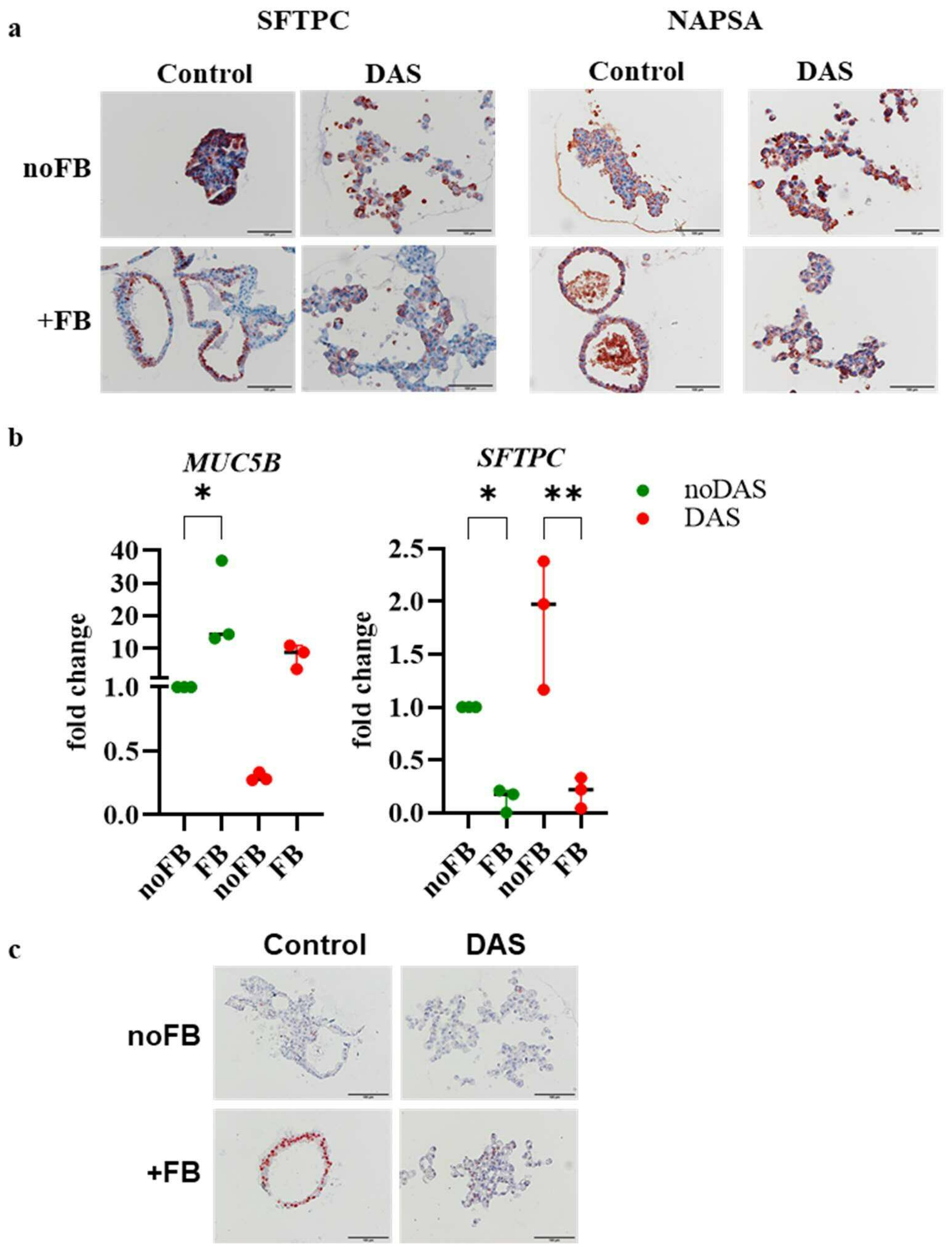

S13. The organoid cultures were incubated with dasatinib (200 nM) from the day of seeding. (a) Immunohistochemistry was performed for SFTPC and NAPSA (scale bar = 100 µm). (b) The expression of SFTPC and MUC5B was confirmed by semi-quantitative RT-PCR. Each data point represents an independent experiment. Data were compared by two-way ANOVA., *p < 0.05, **p < 0.01. (c) Immunohistochemistry was performed for P-STAT3 (scale bar = 100 µm).

Table 1

Primer	Sequence
Human *SFTPC*	Forward: 5'- GCA AAG AGG TCC TGA TGG AG-3'<br>Reverse: 5'- TGT TTC TGG CTC ATG TGG AG-3'
Human *ACTA2*	Forward: 5'- CTATGCCTCTGGACGCACAACT-3'<br>Reverse: 5'- CAGATCCAGACGCATGATGGCA-3'
Human *CTHRC1*	Forward: 5'- CAGGACCTCTTCCCATTGAAGC-3'<br>Reverse: 5'- GCAACATCCACTAATCCAGCACC-3'
Human *MUC5B*	Forward: 5'- CTGCTACGACAAGGACGGAAAC-3'<br>Reverse: 5'- AAGGCTGTGAGCGCACTGGATG-3'
Human *GAPDH*	Forward: 5'- GTCTCCTCTGACTTCAACAGCG -3'<br>Reverse: 5'- ACCACCCTGTTGCTGTAGCCAA -3'

1. **Habermann AC, Gutierrez AJ, Bui LT, Yahn SL, Winters NI, Calvi CL, Peter L, Chung MI, Taylor CJ, Jetter C, Raju L, Roberson J, Ding G, Wood L, Sucre JMS, Richmond BW, Serezani AP, McDonnell WJ, Mallal SB, Bacchetta MJ, Loyd JE, Shaver CM, Ware LB, Bremner R, Walia R, Blackwell TS, Banovich NE, and Kropski JA**. Single-cell RNA sequencing reveals profibrotic roles of distinct epithelial and mesenchymal lineages in pulmonary fibrosis. *Sci Adv* 6: eaba1972, 2020.
